# Effectiveness of school-based child sexual abuse intervention among school children in the new millennium era: Systematic review and meta-analyses

**DOI:** 10.3389/fpubh.2022.909254

**Published:** 2022-07-22

**Authors:** Ruhana Che Yusof, Mohd Noor Norhayati, Yacob Mohd Azman

**Affiliations:** ^1^Department of Family Medicine, School of Medical Sciences, Universiti Sains Malaysia, Kubang Kerian, Malaysia; ^2^Medical Practice Division, Ministry of Health, Level 7, Block E1, Parcel E, Federal Government Administrative Centre, Putrajaya, Malaysia

**Keywords:** school-based intervention, child sexual abuse, knowledge, skills, attitude

## Abstract

**Introduction:**

School-based child sexual abuse intervention programs were developed to educate the school children to protect them from sexual abuse. The programs were evaluated to make sure the interventions were effective in reducing child sexual abuse cases (CSA). This review aimed to determine the effectiveness of the school-based child sexual abuse intervention programs in the new millennium era (2000–2021) in improving the knowledge, skills, and attitude of school children under 18 years old toward child sexual abuse.

**Methods:**

A systematic search was conducted through MEDLINE (PubMed), EBSCO, and SCOPUS databases to collect full English articles related to school-based CSA intervention programs published from 2000 to 2021.

**Results:**

A total of 29 studies from randomized control trial and quasi-experimental from several countries was analyzed. Comparisons within group of pre-post intervention for knowledge, skills, and attitude were measured by standardized mean difference (SMD) and 95% CI of −1.06 (95% CI: −1.29, −0.84), −0.91 (95% CI: −1.2, −0.61), and −0.51 (95% CI: −3.61, 0.58), respectively. Meanwhile for between intervention and control group comparisons, the SMD of knowledge was 0.9 (95% CI: 0.63, 1.18), skills was 0.39 (95% CI: 0.07, 0.71), and attitude was 1.76 (95% CI: 0.46, 3.07).

**Conclusion:**

The programs were found to be effective in improving the knowledge, skills, and attitude of the students from pre-intervention to post-intervention and between the intervention and control groups.

**Systematic Review Registration:** www.crd.york.ac.uk/prospero/display_record.php?ID=CRD42022312383, identifier: CRD42022312383.

## Introduction

Child sexual abuse (CSA) is associated with the risk of adverse psychosocial and health effects, resilience processes include several protective factors that can be enhanced through preventative and early intervention efforts (family support, parent-child relationships, social support, etc.) (1). The World Health Organization has classified CSA as “the involvement of a child in sexual activity that he or she does not fully comprehend, is unable to give informed consent to, or for which the child is not developmentally prepared and cannot give consent, or that violate the laws or social taboos of society” (2).

A previous review (3), had reported the definition of child sexual abuse can be found in the perspective of child protection (ensuring a child's safety), criminal (securing prosecutions), and clinical (the impact of abuse on the child) (4) and classified an act of abuse into three levels which are noncontact, contact, and penetrative abuse. The review also mentioned more recent definitions by a study (5) that incorporate what were seen as recent developments (e.g., peer abuse, child prostitution, internet pornography, pedophile networks, and grooming over the internet), in addition to traditional categories such as incest.

However, the term “child sexual abuse” is defined differently by epidemiological studies, policy documents, and legal frameworks that use different approaches to the composition of the CSA, the definition of the acts that make up the CSA, and the nature of consent. The study developed a conceptual model to classify CSA. According to the model, CSA required the presence of all four factors: (i) the person must be a child; (ii) true consent must be absent; (iii) the acts must be sexual; (iv) the acts must constitute abuse. These definitions of CSA and abuse distinguish the overall idea of CSA from others like assault, harassment, and victimization, and the model demonstrates when and why an act or experience is more properly characterized as CSA (6).

In order to prevent CSA, many intervention programs were made globally. Educate young children on CSA is one approach in the intervention program with assumptions that children can (i) recognize the characteristics of an exploitative or abusive encounter, touch, engagement, or scenario; (ii) psychologically oppose an abuser's threats or manipulations; (iii) defy the authority of an adult; (iv) refuse to accept the abuser's affection, attention, and/or material rewards; (v) be willing to disclose abuse perpetrated by others (7).

The fundamental purpose of CSA intervention efforts has been to change children's knowledge and skills through group-based personal safety instruction, which is frequently offered in educational settings (8). School-based programs can be implemented universally at a minimal cost without stigmatizing people at higher risk, program content corresponds with school health curricula, and schools serve as a direct link to additional preventative targets such as school staff, parents, relatives, and communities (9).

A selection of intervention programs that achieved four or more outcome improvements (e.g., knowledge, skills, emotions, disclosure, and maintenance of gains) was used to identify the essential characteristics of effective intervention programs (3) such as improved conceptual knowledge of sexual abuse (remembering), enhanced detection of likely sexual abuse circumstances (recognition), increased personal safety skill knowledge and sensation of security over personal body area (resisting), and potential and occurrence of disclosures (reporting) (10).

The main focus of school-based CSA programs is to reduce the risk of child abuse by teaching children child abuse-related knowledge and self-protection skills (11). Beyond that, these programs also improved the behavior of participants as well as a positive attitude regarding CSA (12). Thus, this study aimed to review the effectiveness of the school-based CSA intervention programs which specifically focus on CSA on knowledge, skills, and attitude of students under 18 years old in reducing risks of child abuse.

## Methods

### Types of studies

The effectiveness of school-based CSA intervention programs among school students under the age of 18 was evaluated by a systematic review and meta-analysis of research. The outcome measures involved knowledge, skills, and attitude of the children on CSA. The studies were reviewed using the Preferred Reporting Items for Systematic Reviews and Meta-Analyses (PRISMA) 2020 guidelines (13).

### Search methods

A systematic search was conducted to identify relevant articles to include in the review. Databases involved were MEDLINE (PubMed), EBSCO, and SCOPUS based on the search terms “[(school-based) AND (prevention OR intervention)] AND (sexual abuse).” All studies published from the year 2000 to 22 February 2022 were extracted to determine their eligibility for inclusion in this review. The search was limited to full-text articles written in English and the age limit was 18 years old and below. The reference lists of included citations were cross-checked to locate other potentially acceptable research.

### Study selection

All the records found by the search approach were exported to EndNote X8 software (Clarivate Analytics, Philadelphia, PA). Duplicate articles were removed. The automation tool using terms (review) or (prevalence) or (protocol) or (qualitative) or (“meta-analysis”) or (“case report”) was used to remove irrelevant studies by the methodologies. Meanwhile, terms (community) or (alcohol) or (drug) or (“substance use”) or (smoking) or (suicidal) or (HIV or AIDS) or (bullying) or (trauma) or (dating) were used to remove irrelevant studies by the interesting outcome. The titles and abstracts of the identified papers were checked by two independent reviewers (RCY, MNN). To assess their eligibility, the full texts of eligible papers were obtained and thoroughly examined. After a consensus discussion, a third reviewer (YMA) was consulted in the event of a conflict between the two reviewers. The search approach was depicted in the PRISMA flow chart, which included and excluded studies as well as the grounds for exclusion.

### Data extraction and management

The extracted data were entered into Microsoft Excel (Microsoft Corporation, Redmond, WA). The data included the first author, year of publication, study location, study design, study population, sample size, name of the intervention program, tools, outcome measures, and data to generate effect estimates if plausible. The studies with incomplete data were excluded from the review. This review was focused on school children aged under 18 years old with the programs specifically on child sexual abuse only. The interest outcomes of knowledge, skills, and attitude on CSA were measured in this review.

### Assessment of risk of bias

Risk of bias assessment for data quality was performed using the Revised Cochrane risk-of-bias tool for randomized trials (RoB 2) version of 22 August 2019 (14) and Risk Of Bias In Non-randomized Studies - of Interventions (ROBINS-I) (15). Independent bias assessments were carried out by two reviewers (RCY, MNN).

### Measures of treatment effect

The evaluation of the program's effects was reported in pooled standardized mean differences (SMD) of the outcomes (knowledge, skills, and attitude) with a 95% CI. The SMD was used when the studies assess the same outcome but measure it with different tools or scales. The evaluation involved the pre- and post-intervention that measures within-group and comparison between the intervention and control groups that measures between-group effect. Subgroup analysis was done when applicable.

### Data synthesis

Studies with the randomized control trial (RCT) or quasi-experimental pre-post trial were selected to include in the review. Measurement of outcomes between pre- and post-intervention (within-group), and between intervention and control groups (between-group) were extracted from the studies and were recorded in Microsoft Excel (Microsoft Corporation, Redmond, WA).

For pre-post intervention outcome, mean and pooled standard deviation (SD) was used to calculate the SMD for within-group comparison. Meanwhile, for comparison between intervention and control groups, mean difference (MD) and SD_pooled_ of within-group outcome from each group were used to calculate the SMD of between-group comparison. The SMD used the Hedge' g formula to determine pooled intervention-specific SDs as follows:


$$\begin{array}{l} Hedge^{\prime }g=\;\frac{MD}{SD_{pooled}},\\ \text{where}\;SD_{pooled}=\sqrt{\frac{SD_{1}^{2}+SD_{2}^{2}}{2}}. \end{array}$$

Individual study SMDs are weighted before being aggregated in meta-analysis.

The analysis was performed with Review Manager software version 5.4 (Nordic Cochrane Centre, Copenhagen, Denmark). This software used a Hedges' g formula to calculate SMD. The SMD values range between 0.2 and 0.5 and are regarded as small, values between 0.5 and 0.8 are considered medium, and values >0.8 are considered large (16). Continuous data using generic inverse variance with a random-effects model was applied to pool the effect size by the SMD of the studies' data. The heterogeneity was assessed by *I*^2^ statistic and used the guide as outlined: 0–40% might not be important; 30–60% may represent moderate heterogeneity; 50–90% may represent substantial heterogeneity; 75–100% would be considerable heterogeneity (17). Subgroup analysis was performed based on study designs (RCT and quasi-experimental), school levels (preschool, primary, and secondary), and children's abilities (normal children and children with disability). If there was a possibility of publication bias, a visual assessment of funnel plots and statistical analysis by Egger's test was used. Sensitivity analysis was done to assess outliers in the synthesized results. We assessed the quality of evidence for the outcomes according to the GRADEpro methodology (18) for risk of bias, inconsistency, indirectness, imprecision, and publication bias; classified as very low, low, moderate, or high.

## Results

### Study selection

**Figure 1:**
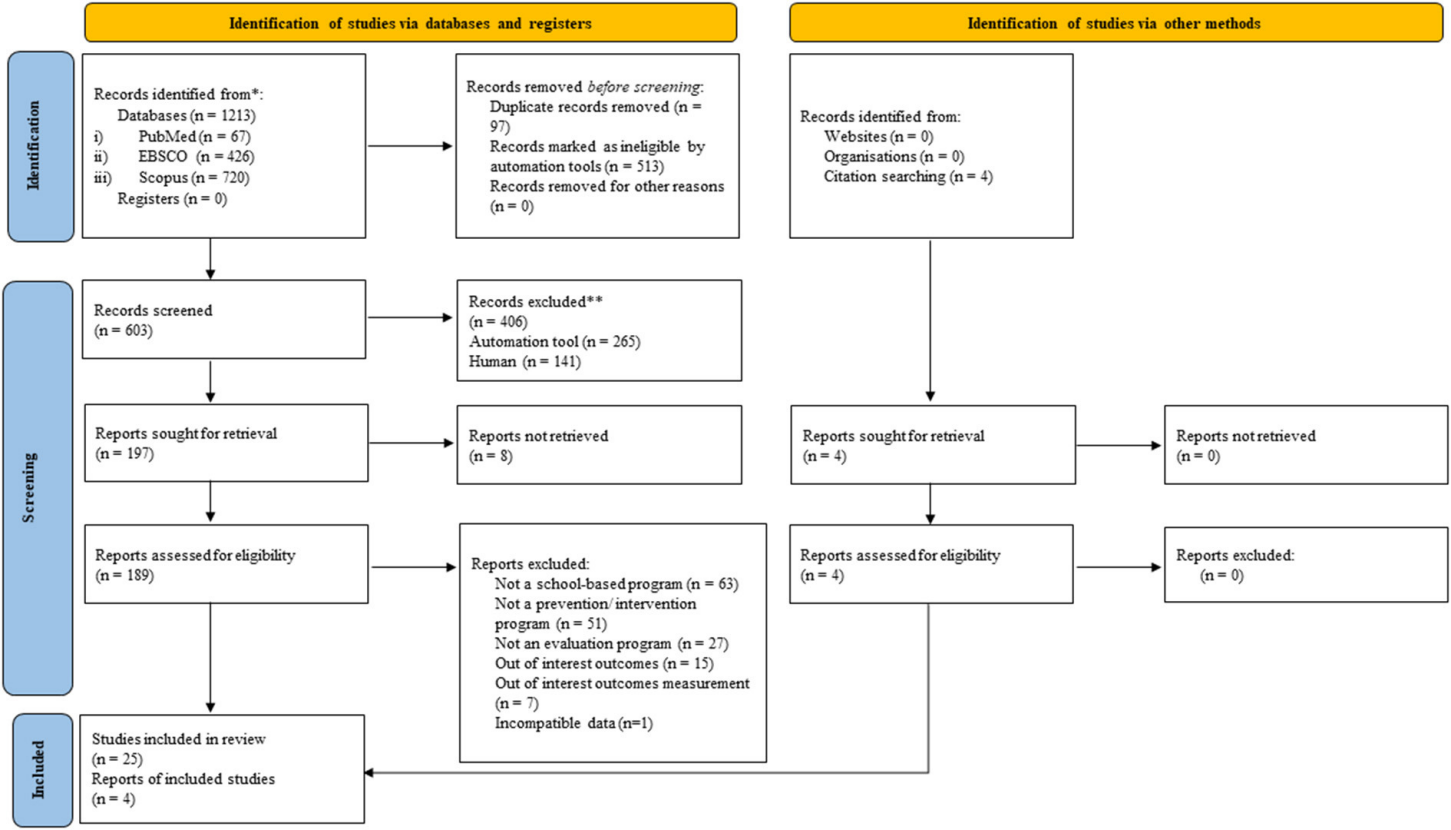
PRISMA 2020 flow chart.

A total of 1,217 studies were identified through the primary search databases and secondary citations. However, 610 studies were removed due to duplicates and were marked as ineligible by automation tools. After screening and retrieving, 189 studies including four studies from citation search were assessed for eligibility. After the assessment, a total of 30 studies met the inclusion and exclusion criteria. However, one study (19) was excluded due to incompatible data and finally, only 29 studies were decided to be included in the review (Figure 1).

### Study characteristics

The included studies were published from the year 2000 to 2021 in the United States (eight studies), Turkey (five studies), Korea (four studies), China (three studies), Germany (three studies), Canada (two studies), and one study each from Spain, Ecuador, Pakistan, and Thailand.

The studies involved 14,817 school children from preschool level (20, 21), primary/elementary school level (23 studies) including a study in special schools for deaf and hard of hearing children, and secondary school level (22, 23). One study was done at both primary and secondary levels (24) and one study was done in a school for children with developmental delays and disorders involving children aged 10 years old to 15 years old (25).

A total of eight studies used RCT as a study design (22, 23, 26–31), and 21 studies by quasi-experimental (20, 21, 24, 25, 32–48). Evaluation of the school-based CSA intervention programs involved the evaluation of knowledge (24 studies), skills (13 studies), and attitude (3 studies) toward CSA. The characteristics of the included studies were summarized in the Supplementary Table 1.

In this new millennium era, some of the intervention programs in this review were developed using new technologies such as web-based and smartphone-based applications (38, 41) since this function is user-friendly due to multi-platform development and can be accessed anytime and anywhere. In 2005, a study in Korea (23) evaluated a CD-ROM-based educational program to increase knowledge of CSA because of its capability to combine audio, visual, and interactive capabilities, and the ability to store text, graphics, images, and movies in a tiny drive. However, in school-based intervention programs, passive teaching strategies such as video, lecture, and workshops, as well as active teaching techniques such as role-playing, modeling, and rehearsing (27, 33), are still used.

Most of the studies in this review measured the outcomes using well-known questionnaires whether in original form or revised or modified versions such as the “Children's Knowledge of Abuse Questionnaire” (CKAQ), the “Personal Safety Questionnaire” (PSQ), “What If” Situation Test (WIST), and the “Body Safety Training Program (BST).” Some of the studies (38, 39, 48) developed new validated questionnaires to measure the outcomes of the studies. The other studies adapted and/or modified the well-known curriculum tools such as the “Good Touch Bad Touch Curriculum Test” (33, 44), “Play it Safe!” (30), “Florida Child Safety Matters®” (CSM) evaluation (31), questionnaire of “Sexuality Knowledge Level” (20) and WHO life skills development concepts questionnaires (24).

### Risk of bias assessment

Data quality assessment for risk of bias was performed using the Revised Cochrane risk-of-bias tool for randomized trials (RoB 2) checklist (14) in eight RCT studies. Meanwhile, the risk of bias assessment by Risk Of Bias In Non-randomized Studies - of Interventions (ROBINS-I) checklist (15) was used in 21 quasi-experimental studies. The overall risk of bias was classified as low risk after the assessment for all the items by two reviewers (RCY, MNN) in both checklists (Supplementary Tables 2, 3).

### Outcomes and subgroup analyses

Table 1 summarized the findings for knowledge, skills, and attitude outcomes for within-group (pre-post intervention) comparison, subgroup analysis by study designs for knowledge outcome, and subgroup analysis by types of children for skills outcome performed. For between-group (intervention and control groups) comparison, subgroup analysis by school levels for knowledge outcome was performed. Subgroup analyses for other outcomes were not performed due to the limited number of studies.

**Table 1:** Comparisons, outcomes, and subgroup analyses.

**Comparison**	**Outcome**	**Subgroup**	**Studies**	***N***	**SMD (95% CI)**	*I*^2^ **(%)**	***p*** **-value**	*I*^2^ **diff (%)**	***p*** **-value**
Within group	Knowledge		24	20,022	−1.06 (−1.29, −0.84)	97	<0.0001		
		Study design						
		RCT	7	6,737	−0.44 (−0.58, −0.31)	81	<0.0001		
		Quasi	17	13,332	−1.43 (−1.78, −1.07)	97	<0.0001		
		Total	24	20,069	−1.06 (−1.29, −0.84)	97	<0.0001	96.1	<0.0001
	Skills		12	4,632	−0.91 (−1.20, −0.61)	94	<0.0001		
		Types of children						
		Normal	10	4,510	−0.76 (−1.04, −0.49)	93	<0.0001		
		With disability	2	156	−4.27 (−10.69, 2.15)	98	<0.0001		
		Total	12	4,666	−0.90 (−1.20, −0.61)	94	<0.0001	7.0	0.300
	Attitude		2	158	−1.51 (−3.61, 0.58)	97	<0.0001		
Between group	Knowledge		20	8,740	0.90 (0.63, 1.18)	97	<0.0001		
		School level							
		Preschool	2	208	3.08 (−0.72, 6.89)	98	<0.0001		
		Primary school	15	7,090	0.84 (0.51, 1.18)	97	<0.0001		
		Secondary school	2	912	0.28 (−0.40, 0.95)	33	0.22		
		Total	19	8,210	0.94 (0.64, 1.24)	97	<0.0001	45.1	0.160
	Skills		13	4,638	0.39 (0.07, 0.71)	95	<0.0001		
	Attitude		3	342	1.76 (0.46, 3.07)	96	0.0002		

*n, number of samples; SMD, standardized mean difference; CI, confidence interval; I^2^, I squared*.

#### Knowledge

The within-group comparison showed that the CSA intervention programs increased knowledge levels in school children compared to pre-intervention [SMD: −1.06 (95% CI: −1.29, −0.84); *I*^2^ = 97%; *p*-value <0.00–1; 24 studies; 20,022 participants] (Table 1) with the SMDs ranging from −0.13 to −7.64 (Supplementary Figure 1).

**Figure 2:**
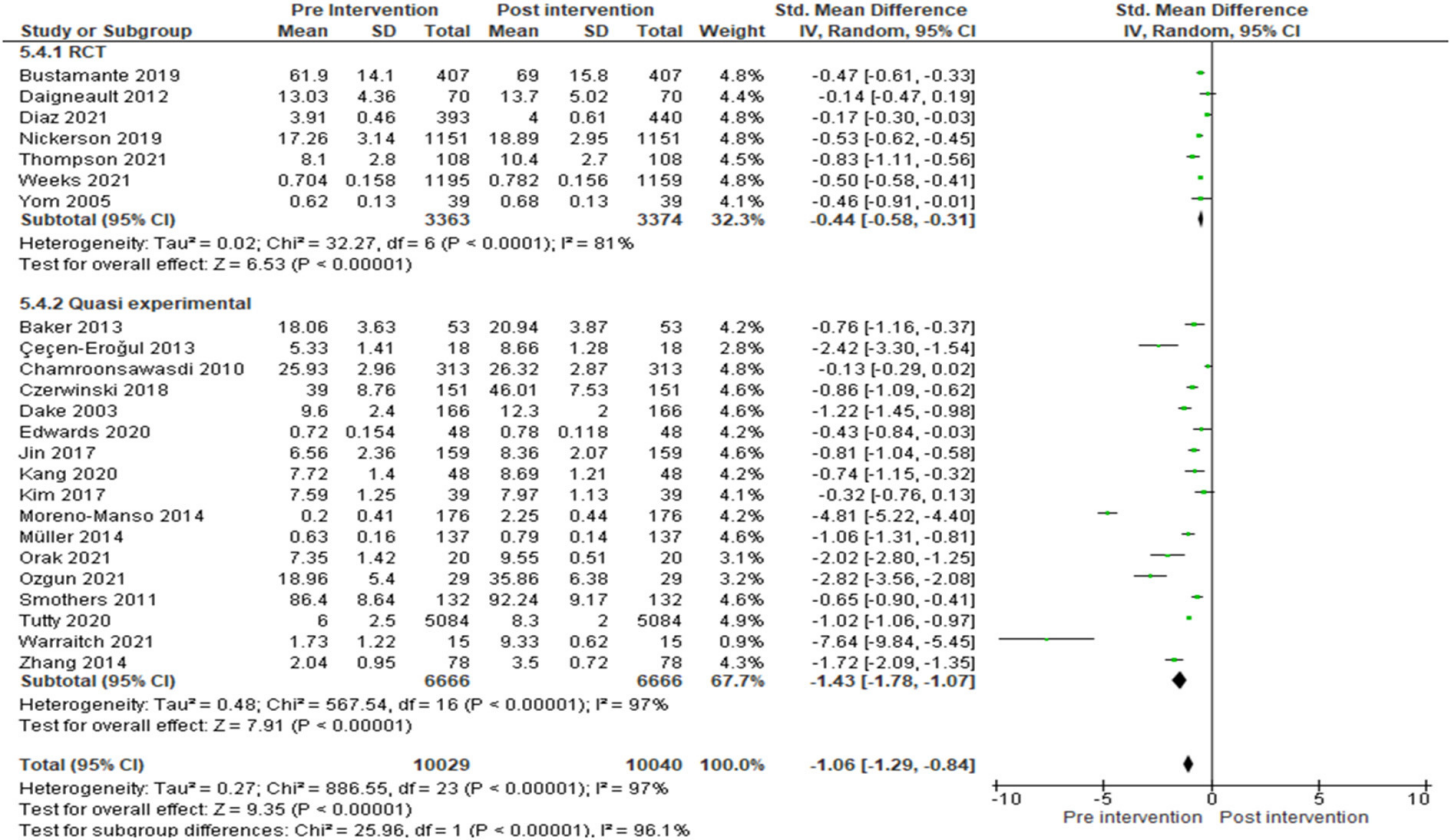
Within-group comparison for knowledge outcome with subgroup analysis by study design.

Subgroup analysis was done by study designs. The knowledge levels in studies with RCT design [SMD: −0.44 [95% CI: −0.58, −0.31]; *I*^2^ = 81%; *p*-value <0.001; seven studies; 6,737 participants; high quality evidence] and quasi-experimental design [SMD: −1.43 (95% CI: −1.78, −1.07); *I*^2^ = 97%; *p*-value <0.0001; 17 studies, 13,332 participant; high quality evidence] (Figure 2, Supplementary Table 4) increased in post-intervention compared to pre-intervention. Heterogeneity was considerable in this subgroup analysis. Large effect size was showed in quasi experimental group but medium effect size in RCT group.

Meanwhile, for between-groups comparison, the CSA knowledge levels were increased in the intervention group compared to the control group [SMD: 0.9 (95% CI: 0.63, 1.18); *I*^2^ = 97%; *p*-value <0.0001; 20 studies; 8,740 participants; high quality evidence] with considerable heterogeneity and large effect size. The SMDs range from −0.33 to 5.06 (Supplementary Figure 2, Supplementary Table 5).

**Figure 3:**
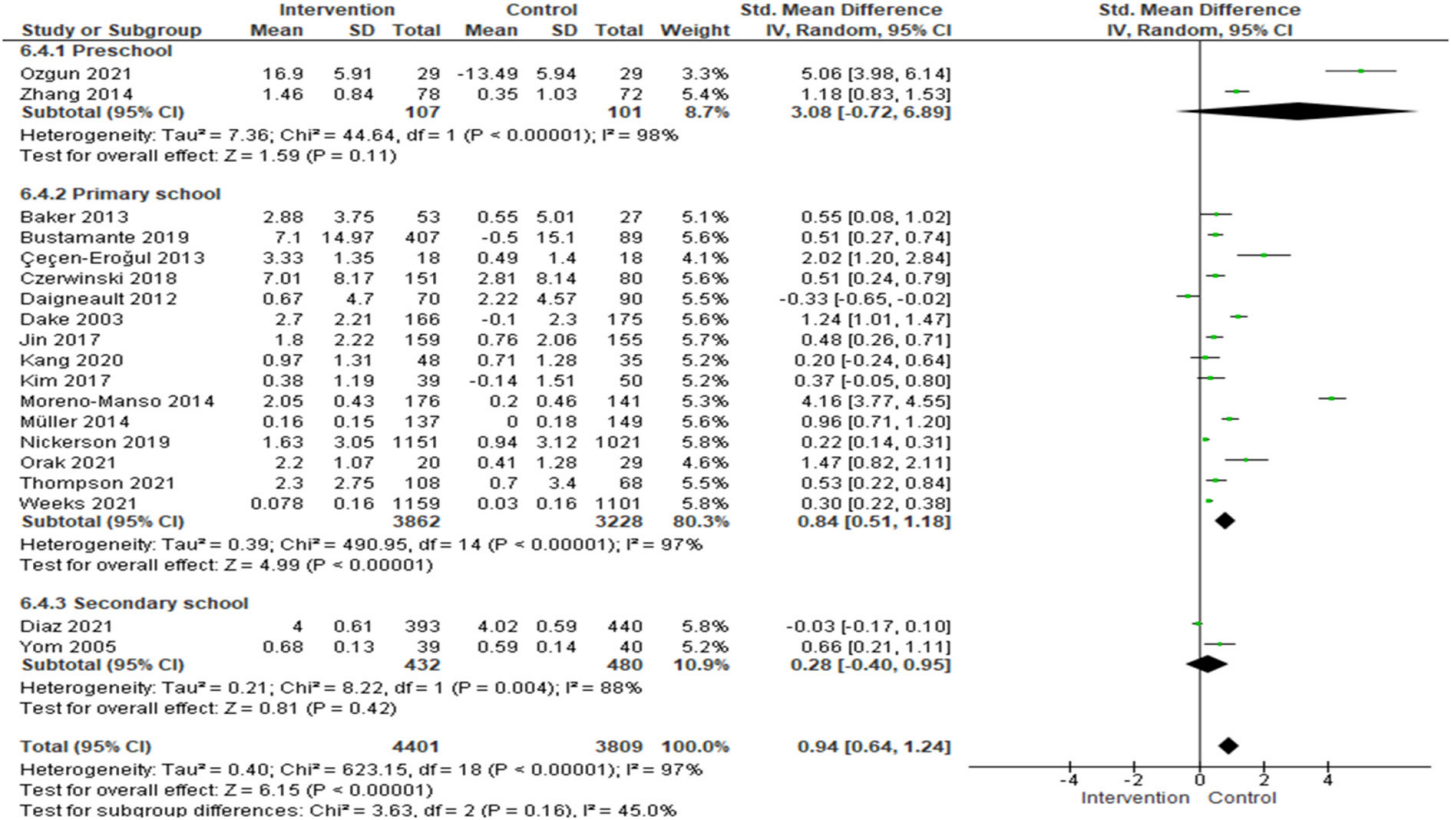
Standardized mean difference of subgroup analysis of school-level by knowledge.

Subgroup analysis by school levels were done for between-group comparison. Subgroup analysis of knowledge levels by, showed that the SMD for preschool were 3.08 [(95% CI: −0.72, 6.89); *I*^2^ = 98%; *p*-value <0.0001; two studies; 208 participants; high quality evidence], for primary school were 0.85 [(95% CI: 0.51, 1.18); *I*^2^ = 97%; *p*-value <0.0001; 15 studies; 7,090 participants; high quality evidence], and for secondary school were 0.28 [(95% CI: −0.4, 0.95); *I*^2^ = 33%; *p*-value = 0.22; two studies; 912 participants; high quality evidence], (Figure 3, Supplementary Table 5). SMD of 15 studies at primary school level showed a large effect size. Considerable heterogeneity was observed in this subgroup analysis.

#### Skills

Within-group comparison showed that the CSA intervention programs increased skills levels among school children in post-intervention compared to pre-intervention [SMD: −0.91 (95% CI: −1.2, −0.61); *I*^2^ = 93%; *p*-value <0.0001; 12 studies; 4,632 participants; Table 1] with large effect size. The SMDs range from −0.14 to −7.64 (Supplementary Figure 3).

**Figure 4:**
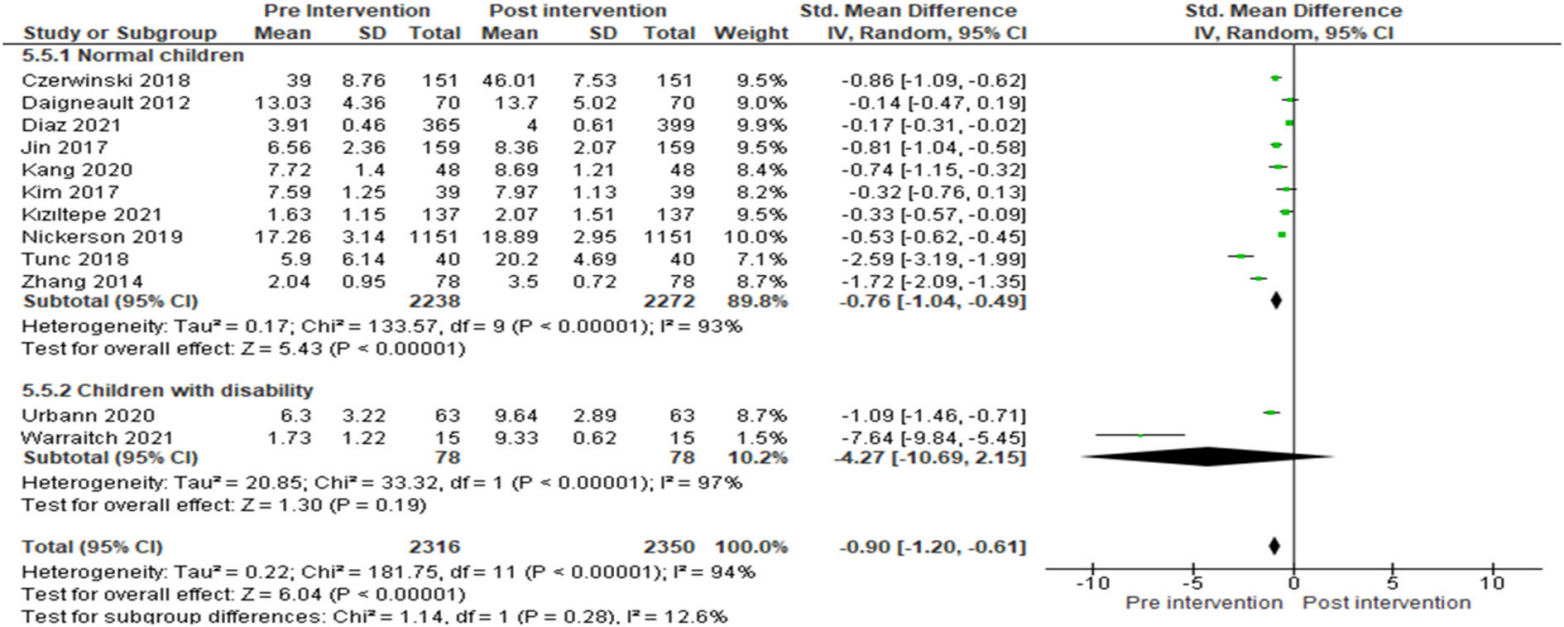
Standardized mean difference of subgroup analysis of the type of children by skills.

Subgroup analysis was done for the type of children in skills outcome. The skills levels was increased in post-intervention compared to pre-intervention in school children with normal ability [SMD: −0.76 (95% CI: −1.04, −0.49); *I*^2^ = 93%; *p*-value <0.0001; 10 studies; 4,510 participants; high quality evidence] and in school children with disability [SMD: −4.27 (95% CI: −10.69, 2.15); *I*^2^ = 98%; *p*-value <0.0001; two studies; 156 participants; high quality evidence; Figure 4, Supplementary Table 4]. The effect size for children with normal ability was considered medium. Considerable heterogeneity was presented in this subgroup analysis.

Meanwhile, between-group comparison showed that the skills levels of CSA were increased in the intervention group compared to the control group [SDM: 0.39 (95% CI: 0.07, 0.71); *I*^2^ = 95%; *p*-value <0.0001; 13 studies; 4,638 participants; Table 1] with considerable heterogeneity and medium effect size. The SMDs range from −1.84 to 3.04 (Supplementary Figure 4). Subgroup analysis was not done for this comparison since the effect size of this outcome was small (SMD <0.5) compared to the other outcomes in which the effect sizes were more than 0.9.

#### Attitude

Within-group comparison of attitude showed that the attitude levels were increased in post-intervention compared to pre-intervention, but the difference was not significant [SMD: −0.51 (95% CI: −3.61, 0.58); *I*^2^ = 97%; *p*-value < 0.0001; two studies; 158 participants; high quality evidence; Supplementary Figure 5, Supplementary Table 4] considerable heterogeneity.

For between-group comparison, the CSA intervention programs increased the attitude level in the intervention group compared to the control group [SMD: 1.76 (95% CI: 0.46, 3.07); *I*^2^ = 96%; %; *p*-value = 0.0002; 3 studies; 342 participants; high quality evidence; Supplementary Figure 6, Supplementary Table 5] considerable heterogeneity. Both models showed considerable heterogeneity. Subgroup analysis was done on this outcome due to a small number of studies.

### Publication bias

Funnel plot asymmetry was observed in within-group and between-group for knowledge and skills outcomes (Supplementary Figures 7–13). For within-group comparison, the Egger's tests were not significant in CSA knowledge levels *(p*-value = 0.583) and in study designs assessment *(p*-value = 0.581) but significant in skills levels *(p*-value = 0.043) and type of children (*p*-value = 0.042).

Meanwhile for between-group comparison, the Egger's tests were significant in knowledge levels (*p*-value = 0.012) and school levels *(p*-value = 0.014) but not significant in skills levels (*p*-value = 0.58). Publication bias was not assessed for attitude outcome due to a limited number of studies.

### Outlier and sensitivity analysis

Finally, one study (25) was detected as an outlier because its values were beyond the range of values compared to other studies. It was included in the within-group analyses of knowledge levels, subgroup analyses of quasi-experimental (study design) and skills levels, and in the subgroup of children with disability (type of children).

Sensitivity analysis for the within-group comparison for skills levels outcome showed a change in the pooled estimate of SMD from −6.6 (95% CI: (−17.66, 4.47) to −1.09 (95% CI: (−1.46, −0.71) for children with disability. There were no differences in the effect estimates for within-group for knowledge levels, within-group comparison of study design subgroup for the quasi-experimental and total subgroup, within-group skills levels, and the total subgroup of type of children (Table 2).

**Table 2:** Sensitivity analysis for an outlier study (25).

**Comparison**	**Outcome**	**Subgroup**	***N*** **studies**	**Pooled estimate with**	***I***^2^ **(%)**	***N*** **studies**	**Pooled estimate without**	***I***^2^ **(%)**
				**outlier SMD^a^ (95% CI)**			**outlier SMD^a^ (95% CI)**	
**Within group**	**Knowledge**		**24**	**−1.06 (−1.29, −0.84)**	**97**	**23**	**−1.01 (−1.22, −0.79)**	**97**
		**Study design**					
		**Quasi**	**17**	**−1.43 (−1.78, −1.07)**	**97**	**16**	**−1.31 (−1.65, −0.96)**	**97**
		**Total**	**24**	**−1.06 (−1.29, −0.84)**	**97**	**23**	**−1.01 (−1.22, −0.79)**	**97**
	**Skills**		**12**	**−0.87 (−1.17, −0.57)**	**94**	**11**	**−0.77 (−1.04, −0.51)**	**93**
		**Type of children**					
		**With disability**	**2**	**−6.6 (−17.66, 4.47)**	**98**	**1**	**−1.09 (−1.46, −0.71)**	**NA**
		**Total**	**12**	**−0.87 (−1.16, −0.57)**	**94**	**11**	**−0.79 (−1.06, −0.53)**	**93**

a *Standardized mean difference*.

## Discussion

This review found that the school-based CSA intervention programs from various studies between the year 2000 to 2021 were effective in increasing knowledge, skills, and attitude toward CSA among school children aged under 18 years old. Further explorations by subgroup analyses found that the SMDs were higher quasi-experimental for study design, and in children with disability in the type children. At the school level, preschool children had the highest SMD compared to primary and secondary school children which indicates the age difference in knowledge levels following the CSA intervention programs. However, the conclusion should be made cautiously due to the heterogeneity in the analyses.

The most often assessed study outcome on the effectiveness of the CSA intervention programs was knowledge acquisition, either through questionnaires aimed to capture factual knowledge or through vignettes that attempted to determine applied knowledge (49). In this review, all studies showed an increasing knowledge of CSA after the intervention programs. A meta-analysis showed that the CSA intervention programs gained factual and applied knowledge as early up to two weeks after the intervention. Older children appeared to gain better knowledge than younger children using questionnaire-based measures compared to vignette-based measures (49). A review reported that preschool children had a larger increase in CSA knowledge than elementary and middle school students (50). However, we found no difference in CSA knowledge in preschool, primary and secondary schools. Considerable heterogeneity was present with only two studies representing preschool level and secondary school level. A large effect size was also presented in the overall school-level model. A recent review reported that CSA-related knowledge had a significant overall effect with a medium effect size on school-based CSA intervention programs after controlling the moderating effects of samples, study designs, and program characteristics by two three-level meta-analyses (51).

Subgroup analyses by study design reported medium effect size in seven RCTs and large effect size in 17 quasi-experimental studies. A well-designed RCT provided strong evidence of a cause-effect relation in evaluating the intervention and the study design was also capable of determining the validity and generalizability of the findings (52) compared to quasi-experimental. A quasi-experimental of nonrandomized study or pre-post intervention study was chosen due to the small availability of participants in the included studies. Eleven quasi-experimental studies in our review had less than 100 sample sizes. Other reasons for the use of this design are when randomization is not allowed for known efficacy intervention and difficulty in randomizing the participants and the locations (53).

The CSA intervention programs also evaluated the skills of children. The differences were seen in both comparisons, pre-post intervention and between intervention and control group. The effect on skills was similar to a review in China (50). Generally, the important components of self-protection skills that are included in intervention programs were to disclose abuse, recognized risk situations, deal with emotions, get away and find help, assertiveness skills to say “no,” and increase self-esteem (54). The likelihood of sexual abuse is higher in children with disability compared to normal children and higher in certain types of disabilities such as mental or intellectual disability (55). The effectiveness of intervention programs for children with intellectual disabilities should be adapted based on the needs of the children and learning styles such as simplifying words or symbolic communication such as pictures, images, symbols, and signs with more repetition since the children are known to have lacked in verbal skills (56).

Evaluation of attitude or perception or belief was one of the components in evaluating a CSA intervention program. A good positive attitude toward personal safety is required to face sexual abuse (46) and all the involved studies in this review showed increased attitudes towards child sexual abuse both within and between groups comparisons. A review (57) stated that the participants used the knowledge and skills they had gained from the intervention programs in specific real-life circumstances and were able to help friends with the information (58) The attitude toward sexual abuse may influence children's behavior and the ability of a person to respond to the sexual abuse (59). The greater impact, especially for preschool children may be achieved by integrating entertainment, such as videos, songs, and picture books in the intervention programs (28). It potentially improved the attitude of the children toward sexual abuse (60).

Funnel plot asymmetry should not be confused with publication bias because it might have a variety of other reasons (61). The asymmetry shapes of funnel plots in this review were believed to arise from the heterogeneity of the study. One approach to account for heterogeneity is assuming that heterogeneity is random by a random-effect model. Asymmetry or unusual shapes in funnel plots can be caused by heterogeneity, reporting bias, or chance (61). Asymmetry of funnel plots was measured by Egger's test which is based on regression intercept. Egger's test reports the *p*-value of the regression but not the magnitude of the intercept due to difficulty in providing a severity range of publication bias (62). Publication bias is difficult to assess in reviews of 10 or fewer studies due to a lack of power and in reviews of non-randomized studies due to confounding concerns (63).

This review had limitations. This review did not apply meta-regression in the analysis. No mediator or moderator effect was analyzed. On the other hand, a study in China showed the mediator effect of attitude towards messages from picture books that mediated the message framing on refusal skills. This in turn inspired participants to apply the refusal skills learned from the messages (28). This review also focused only on CSA and did not include physical abuse, emotional abuse, and neglect. The search was limited to publications published in English exclusively which may have reduced the generalization of this review. Limited database search can introduce publication bias. However, MEDLINE was determined to be the best single source for retrieving a systematic review, with an 89.7% inclusion of free and open-access papers (64). However, it is also advised to do a thorough search for research utilizing multiple databases to decrease potential biases in the included studies.

## Conclusion

The school-based CSA intervention programs held from the year 2000 to 2021 in the new millennium era were reported to increase the knowledge, self-protection skills, and attitude toward CSA among school children under 18 years old. The improvement in the key components of CSA intervention programs indicated that the programs were effective. However, it was not proven to reduce the risk of CSA since this review did not study the prevalence of CSA in this duration.

## Data availability statement

The original contributions presented in the study are included in the article/supplementary material, further inquiries can be directed to the corresponding author.

## Author contributions

RCY and MNN: conceptualization, methodology, and validation. RCY: software, formal analysis, data curation, and writing—original draft preparation. MNN and YMA: writing—review and editing. MNN: visualization and supervision. All authors have read and agreed to the published version of the manuscript.

## Funding

This work was funded by Fundamental Research Grant Scheme, Higher Education Department, Ministry of Education. Malaysia-Framework for sex abuse incest and SmartShield sex abuse prevention in primary school children FRGS/1/2020/SS0/USM/02/12 (203.PPSP.6171295).

## Conflict of interest

The authors declare that the research was conducted in the absence of any commercial or financial relationships that could be construed as a potential conflict of interest. The reviewer SA declared a shared affiliation with the author YMA to the handling editor at the time of review.

## Publisher's note

All claims expressed in this article are solely those of the authors and do not necessarily represent those of their affiliated organizations, or those of the publisher, the editors and the reviewers. Any product that may be evaluated in this article, or claim that may be made by its manufacturer, is not guaranteed or endorsed by the publisher.

## Supplementary material

Supplementary Figure 1 Standardized mean difference of within-group intervention by knowledge.

Supplementary Figure 2 Standardized mean difference of between-group intervention by knowledge.

Supplementary Figure 3 Standardized mean difference of within-group intervention by skills.

Supplementary Figure 4 Standardized mean difference of between-group intervention by skills.

Supplementary Figure 5 Standardized mean difference of within-group intervention by attitude.

Supplementary Figure 6 Standardized mean difference of between-group intervention by attitude.

Supplementary Figure 7 Funnel plot of within-group comparison by knowledge.

Supplementary Figure 8 Funnel plot of study design by knowledge.

Supplementary Figure 9 Funnel plot of within-group comparison by skills.

Supplementary Figure 10 Funnel plot of type of children by skills.

Supplementary Figure 11 Funnel plot of between-group comparison by knowledge.

Supplementary Figure 12 Funnel plot of school level by knowledge.

Supplementary Figure 13 Funnel plot of between-group comparison by skills.

Supplementary Table 1 Characteristics of the included study.

Supplementary Table 2 Assessment risk of bias by ROB-2.

Supplementary Table 3 Assessment risk of bias by ROBINS-I.

Supplementary Table 4 The quality of evidence for the within-group comparison according to GRADEpro methodology.

Supplementary Table 5 The quality of evidence for the between-group comparison according to GRADEpro methodology.

## References

[1] Murray LK Nguyen A Cohen JA. Child sexual abuse. Child Adolesc Psychiatr Clin N Am. (2014) 23:321–37. 10.1016/j.chc.2014.01.003 24656583 PMC4413451

[2] WHO. Report of the Consultation on Child Abuse Prevention. WHO/HSC/PVI/99.1. World Health Organization. Geneva: WHO (1999).

[3] Topping KJ Barron IG. School-based child sexual abuse prevention programs: a review of effectiveness. Rev Educ Res. (2009) 79: 431–63. 10.3102/003465430832558225528978

[4] Faller KC. Child Sexual Abuse: Intervention and Treatment Issues. Washington, DC: Services UDoHaH. Circle, Inc., McLean, VA: Westover Consultants, Inc. (1993). 10.1037/e624552007-001

[5] Chase E Statham J. Commercial and sexual exploitation of children and young people in the UK—a review. Child Abus Rev. (2005) 14:4–25. 10.1002/car.881

[6] Mathews B Collin-Vézina D. Child sexual abuse: toward a conceptual model and definition. Trauma Violence Abuse. (2017) 20:131–48. 10.1177/1524838017738726 29333990 PMC6429628

[7] Rudolph J Zimmer-Gembeck MJ Shanley DC Hawkins R. Child sexual abuse prevention opportunities: parenting, programs, and the reduction of risk. Child Maltreat. (2017) 23:96–106. 10.1177/1077559517729479 28920456

[8] Wurtele SK. Preventing sexual abuse of children in the twenty-first century: preparing for challenges and opportunities. J Child Sex Abus. (2009) 18:1–18. 10.1080/10538710802584650 19197612

[9] Walsh K Zwi K Woolfenden S Shlonsky A. School-based education programmes for the prevention of child sexual abuse. Cochrane Database Syst Rev. (2015) 4:Cd004380. 10.1002/14651858.CD004380.pub3 25876919 PMC9805791

[10] Heidotting T Keiffer S Soled S. A quantitative synthesis of child sexual abuse prevention programs. In: The Anual Meeting of The American Educational Research Association. New Orleans, LA: The American Educational Research Association (1994). Available online at: https://files.eric.ed.gov/fulltext/ED376217.pdf (accessed March 22, 2022).

[11] Blakey JM Thigpen JW. Play it Safe!®: a school-based childhood physical and sexual abuse prevention program. J Adoles and Fam Health. (2015) 7:1. 10.1016/j.chiabu.2019.104092 31425883

[12] Martin EK Silverstone PH. An evidence-based education program for adults about child sexual abuse (“Prevent It!”) that significantly improves attitudes, knowledge, and behavior. Front Psychol. (2016) 7:1177. 10.3389/fpsyg.2016.01177 27594844 PMC4991113

[13] Page MJ McKenzie JE Bossuyt PM Boutron I Hoffmann TC Mulrow CD . The PRISMA 2020 statement: an updated guideline for reporting systematic reviews. BMJ. (2021) 372:n71. 10.1136/bmj.n71 33782057 PMC8005924

[14] Sterne JAC Savović J Page MJ Elbers RG Blencowe NS Boutron I . RoB 2: a revised tool for assessing risk of bias in randomised trials. BMJ. (2019) 366:l4898. 10.1136/bmj.l4898 31462531

[15] Sterne JA Hernán MA Reeves BC Savović J Berkman ND Viswanathan M . ROBINS-I: a tool for assessing risk of bias in non-randomised studies of interventions. BMJ. (2016) 355:i4919. 10.1136/bmj.i4919 27733354 PMC5062054

[16] Andrade C. Mean difference, standardized mean difference (SMD), and their use in meta-analysis: as simple as it gets. J Clin Psychiatry. (2020) 81:5. 10.4088/JCP.20f13681 32965803

[17] Higgins J Thomas J Chandler J Cumpston M Li T Page M . Cochrane handbook for systematic reviews of interventions version 6.2. Cochrane. (2021). Available online at: www.training.cochrane.org/handbook (accessed March 22, 2022).

[18] Guyatt GH Oxman AD Kunz R Vist GE Falck-Ytter Y Schünemann HJ. What is “quality of evidence” and why is it important to clinicians? BMJ. (2008) 336:995. 10.1136/bmj.39490.551019.BE 18456631 PMC2364804

[19] Kenny MC Wurtele SK Alonso L. Evaluation of a personal safety program with Latino preschoolers. J Child Sex Abus. (2012) 21:368–85. 10.1080/10538712.2012.675426 22809044

[20] Ozgun SY Capri B. The effect of sexuality education program on the sexual development of children aged 60–72 months. Curr Psychol. (2021) 21:8. 10.1007/s12144-021-02040-8

[21] Zhang W Chen J Feng Y Li J Liu C Zhao X. Evaluation of a sexual abuse prevention education for Chinese preschoolers. Res Soc Work Pract. (2014) 24:428–36. 10.1177/1049731513510409

[22] Diaz MJ Moreland D Wolfersteig W. Assessing the effects of Childhelp's Speak Up be Safe child abuse prevention curriculum for high school students. J Child Adolesc Trauma. (2021) 14:425–32. 10.1007/s40653-021-00353-1 33968290 PMC8090909

[23] Yom YH Eun LK. Effects of a CD-ROM educational program on sexual knowledge and attitude. Comput Inform Nurs. (2005) 23:214–9. 10.1097/00024665-200507000-00009 16027537

[24] Chamroonsawasdi K Suparp J Kittipichai W Khajornchaikul P. Gender roles, physical and sexual violence prevention in primary extend to secondary school in Samutsakorn province, Thailand. J Med Assoc Thai. (2010) 93:358–65. 20420112

[25] Warraitch A Amin R Rashid A. Evaluation of a school-based sexual abuse prevention program for female children with intellectual disabilities in rural Pakistan- a feasibility study. Appl Nurs Res. (2021) 57:151391. 10.1016/j.apnr.2020.151391 33549294

[26] Bustamante G Andrade MS Mikesell C Cullen C Endara P Burneo V . “I have the right to feel safe”: evaluation of a school-based child sexual abuse prevention program in Ecuador. Child Abuse Negl. (2019) 91:31–40. 10.1016/j.chiabu.2019.02.009 30822629

[27] Daigneault I Hébert M McDuff P Frappier JY. Evaluation of a sexual abuse prevention workshop in a multicultural, impoverished urban area. J Child Sex Abuse. (2012) 21:521–42. 10.1080/10538712.2012.703291 22994691

[28] Huang S Cui C. Preventing child sexual abuse using picture books: the effect of book character and message framing. J Child Sex Abus. (2020) 29:448–67. 10.1080/10538712.2020.1719449 32109197

[29] Nickerson AB Tulledge J Manges M Kesselring S Parks T Livingston JA . Randomized controlled trial of the Child Protection Unit: grade and gender as moderators of CSA prevention concepts in elementary students. Child Abuse Negl. (2019) 96:104101. 10.1016/j.chiabu.2019.104101 31377534

[30] Thompson EL Zhou Z Garg A Rohr D Ajoku B Spence EE. Evaluation of a school-based child physical and sexual abuse prevention program. Health Educ Behav. (2021) 2021:1090198120988252. 10.1177/1090198120988252 33605168

[31] Weeks EA Whitaker DJ Pendarvis S Finkelhor D Neal-Rossi C Rivers D. Evaluation of the child safety matters curriculum for improving knowledge about victimization among elementary school children: a randomized trial. J Child Sex Abus. (2021) 2021:1960458. 10.1080/10538712.2021.1960458 34382504

[32] Baker CK Gleason K Naai R Mitchell J Trecker C. Increasing knowledge of sexual abuse: a study with elementary school children in Hawai'i. Res Soc Work Pract. (2013) 23:167–78. 10.1177/1049731512468796

[33] Çeçen-Erogul AR Hasirci ÖK. The effectiveness of psycho-educational school-based child sexual abuse prevention training program on turkish elementary students. Kuram ve Uygulamada Egitim Bilim. (2013) 13:725–9.

[34] Czerwinski F Finne E Alfes J Kolip P. Effectiveness of a school-based intervention to prevent child sexual abuse: evaluation of the German IGEL program. Child Abuse Negl. (2018) 86:109–22. 10.1016/j.chiabu.2018.08.023 30278285

[35] Dake JA Price JH Murnan J. Evaluation of a child abuse prevention curriculum for third-grade students: assessment of knowledge and efficacy expectations. J Sch Health. (2003) 73:76–82. 10.1111/j.1746-1561.2003.tb03576.x 12643023

[36] Edwards KM Siller L Leader Charge L Bordeaux S Leader Charge D Herrington R. Efficacy of a sexual abuse prevention program with children on an Indian reservation. J Child Sex Abus. (2020) 29:900–10. 10.1080/10538712.2020.1847229 33206586

[37] Jin Y Chen J Jiang Y Yu B. Evaluation of a sexual abuse prevention education program for school-age children in China: a comparison of teachers and parents as instructors. Health Educ Res. (2017) 32:364–73. 10.1093/her/cyx047 28854573

[38] Kang SR Kim SJ Kang KA. Effects of child sexual abuse prevention education program using hybrid application (CSAPE-H) on fifth-grade students in South Korea. J Sch Nurs. (2020) 38:1059840520940377. 10.1177/1059840520940377 32691681

[39] Kim SJ Kang KA. Effects of the child sexual abuse prevention education (C-SAPE) program on south Korean fifth-grade students' competence in terms of knowledge and self-protective behaviors. J Sch Nurs. (2017) 33:123–32. 10.1177/1059840516664182 27573418

[40] Kiziltepe R Eslek D Irmak TY Güngör D. “I am learning to protect myself with Mika:”a teacher-based child sexual abuse prevention program in Turkey. J Interpers Violence. (2021) 2021:886260520986272. 10.1177/0886260520986272 33446045

[41] Moon KJ Park KM Sung Y. Sexual abuse prevention mobile application (SAP_MobAPP) for primary school children in Korea. J Child Sex Abus. (2017) 26:573–89. 10.1080/10538712.2017.1313350 28661824

[42] Moreno-Manso JM García-Baamonde E Blázquez-Alonso M Pozueco-Romero JM. Application of a child abuse prevention programme in an educational context. An Psicol. (2014) 30:1014–24. 10.6018/analesps.30.3.154231

[43] Müller AR Röder M Fingerle M. Child sexual abuse prevention goes online: introducing “Cool and Safe” and its effects. Comput Educ. (2014) 78:60–5. 10.1016/j.compedu.2014.04.023

[44] Orak OS Okanli A. The effect of preventive psychosocial interventions directed towards mothers and children on children's knowledge about protection from sexual abuse. J Child Adolesc Psychiatr Nurs. (2021) 2021:jcap.12334. 10.1111/jcap.12334 34057269

[45] Smothers MK Smothers DB. A sexual assault primary prevention model with diverse urban youth. J Child Sex Abus. (2011) 20:708–27. 10.1080/10538712.2011.622355 22126112

[46] Tunc G Gorak G Ozyazicioglu N Ak B Isil O Vural P. Preventing child sexual abuse: body safety training for young children in Turkey. J Child Sex Abus. (2018) 27:347–64. 10.1080/10538712.2018.1477001 29856274

[47] Tutty LM Aubry D Velasquez L. The “Who Do You Tell?”™ Child sexual abuse education program: eight years of monitoring. J Child Sex Abus. (2020) 29:2–21. 10.1080/10538712.2019.1663969 31525148

[48] Urbann K Bienstein P Kaul T. The evidence-based sexual abuse prevention program: strong with Sam. J Deaf Stud Deaf Educ. (2020) 25:421–9. 10.1093/deafed/enaa019 32696964

[49] Walsh K Zwi K Woolfenden S Shlonsky A. School-based education programs for the prevention of child sexual abuse: a Cochrane systematic review and meta-analysis. Res Soc Work Pract. (2018) 28:33–55. 10.1177/1049731515619705

[50] Zhang H Shi R Li Y Wang Y. Effectiveness of school-based child sexual abuse prevention programs in China: a meta-analysis. Res Soc Work Pract. (2021) 31:693–705. 10.1177/10497315211022827

[51] Gubbels J van der Put CE Stams GJM Assink M. Effective components of school-based prevention programs for child abuse: a meta-analytic review. Clin Child Fam Psychol Rev. (2021) 24:553–78. 10.1007/s10567-021-00353-5 34086183 PMC8176877

[52] Kendall JM. Designing a research project: randomised controlled trials and their principles. Emerg Med J. (2003) 20:164. 10.1136/emj.20.2.164 12642531 PMC1726034

[53] Harris AD McGregor JC Perencevich EN Furuno JP Zhu J Peterson DE . The use and interpretation of quasi-experimental studies in medical informatics. J Am Med Inform Assoc. (2006) 13:16–23. 10.1197/jamia.M1749 16221933 PMC1380192

[54] Gubbels J Assink M Prinzie P van der Put CE. What works in school-based programs for child abuse prevention? The perspectives of young child abuse survivors. Soc Sci. (2021) 10:10. 10.3390/socsci10100404

[55] Smith N Harrell S. Sexual Abuse of Children With Disabilities: A National Snap. New York, NY: Vera Institute of Justice (2013).

[56] Stobbe KJ Scheffers M van Busschbach JT Didden R. Prevention and intervention programs targeting sexual abuse in individuals with mild intellectual disability: a systematic review. J Ment Health Res Intellect Disabil. (2021) 14:135–58. 10.1080/19315864.2021.1883780

[57] Finkelhor D Dziuba-Leatherman J. Victimization prevention programs: a national survey of children's exposure and reactions. Child Abuse Negl. (1995) 19:129–39. 10.1016/0145-2134(94)00111-7 7780776

[58] Del Campo A Fávero M. Effectiveness of programs for the prevention of child sexual abuse: a comprehensive review of evaluation studies. Eur Psychol. (2020) 25:1–15. 10.1027/1016-9040/a00037931262233

[59] Ogunfowokan AA Fajemilehin RB. Impact of a school-based sexual abuse prevention education program on the knowledge and attitude of high school girls. J Sch Nurs. (2012) 28:459–68. 10.1177/1059840512446949 22645094

[60] Solehati T Hermayanti Y Kosasih CE Mediani HS. The effect of child sexual abuse prevention song among elementary school children: Pilot study. J Aisyah. (2021) 6:2. 10.30604/jika.v6i2.473

[61] Sterne JAC Sutton AJ Ioannidis JPA Terrin N Jones DR Lau J . Recommendations for examining and interpreting funnel plot asymmetry in meta-analyses of randomised controlled trials. BMJ. (2011) 343:d4002. 10.1136/bmj.d4002 21784880

[62] Lin L Chu H. Quantifying publication bias in meta-analysis. Biometrics. (2018) 74:785–94. 10.1111/biom.12817 29141096 PMC5953768

[63] Dalton JE Bolen SD Mascha EJ. Publication bias: the elephant in the review. Anesth Analg. (2016) 123:812–3. 10.1213/ANE.0000000000001596 27636569 PMC5482177

[64] Goossen K Hess S Lunny C Pieper D. Database combinations to retrieve systematic reviews in overviews of reviews: a methodological study. BMC Med Res Methodol. (2020) 20:3. 10.1186/s12874-020-00983-3 32487023 PMC7268249

