# Supplementary figures and images for: Effectiveness of school-based child sexual abuse intervention among school children in the new millennium era: Systematic review and meta-analyses
Source: Front Public Health. 2022 Jul 22;10:909254. doi: 10.3389/fpubh.2022.909254 (PMC9355675; doi:10.3389/fpubh.2022.909254)

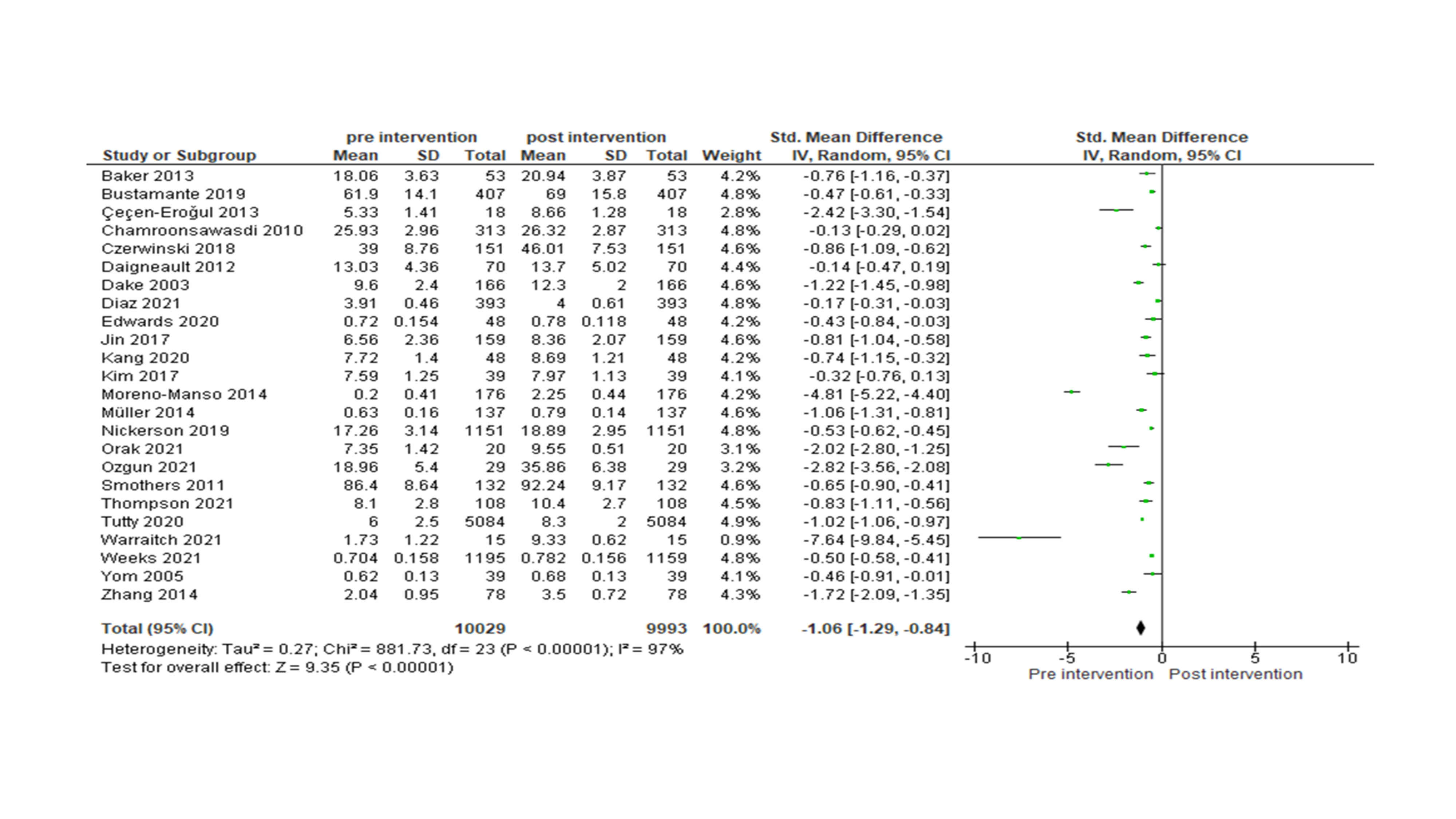

Supplement: Supplementary Figure 1 — Standardized mean difference of within-group intervention by knowledge. [file Image_1.TIF]

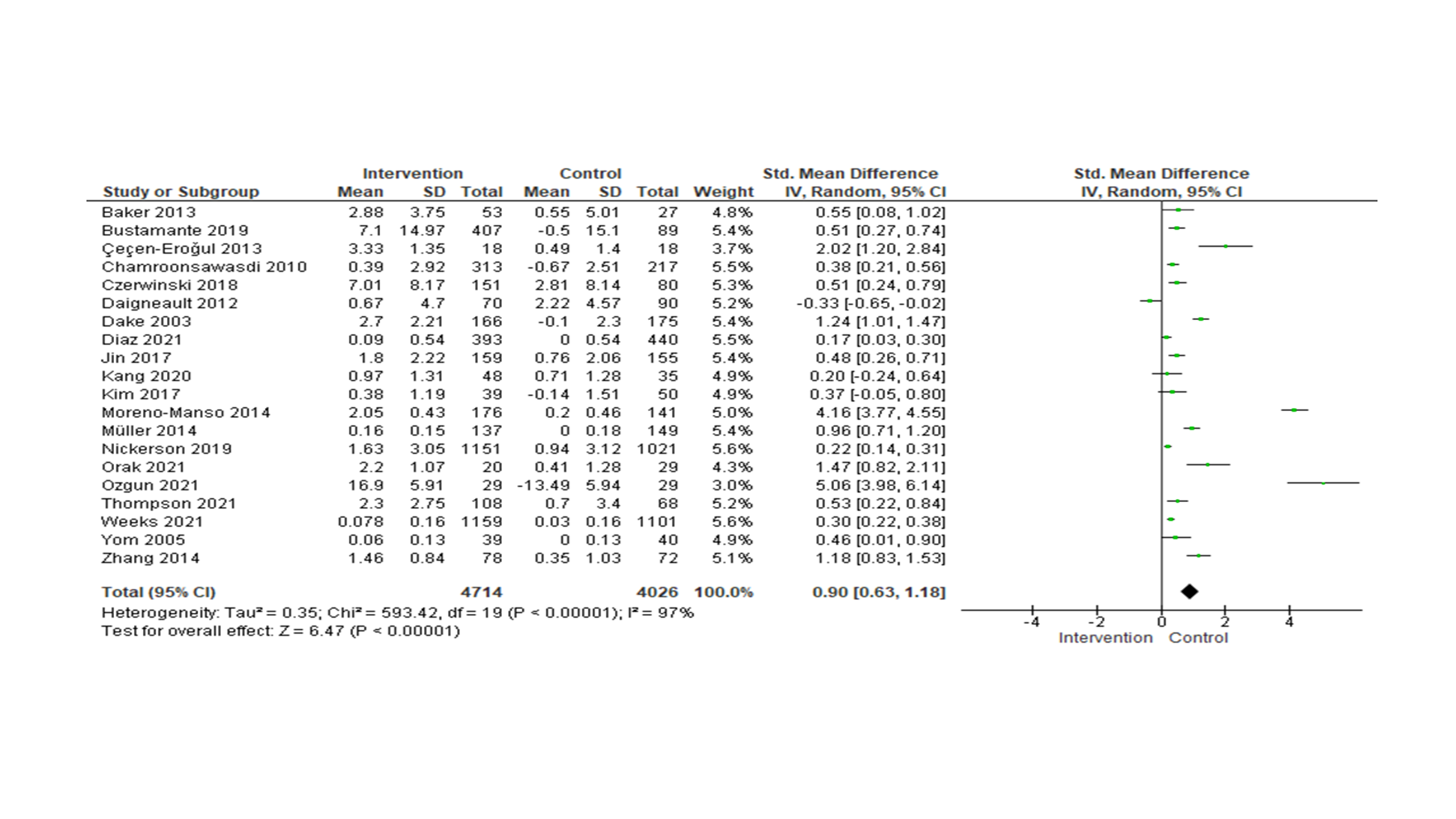

Supplement: Supplementary Figure 2 — Standardized mean difference of between-group intervention by knowledge. [file Image_2.TIF]

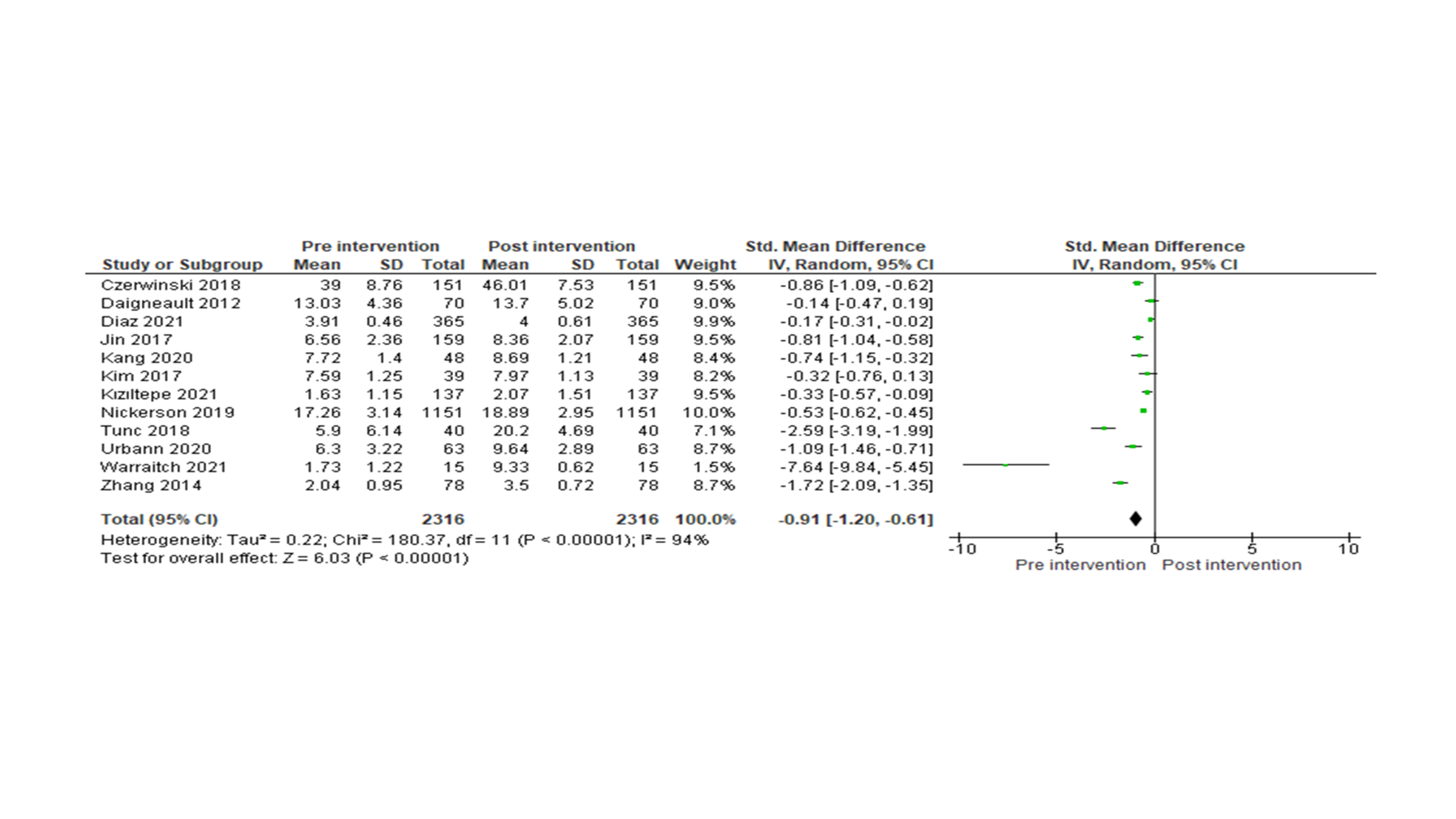

Supplement: Supplementary Figure 3 — Standardized mean difference of within-group intervention by skills. [file Image_3.TIF]

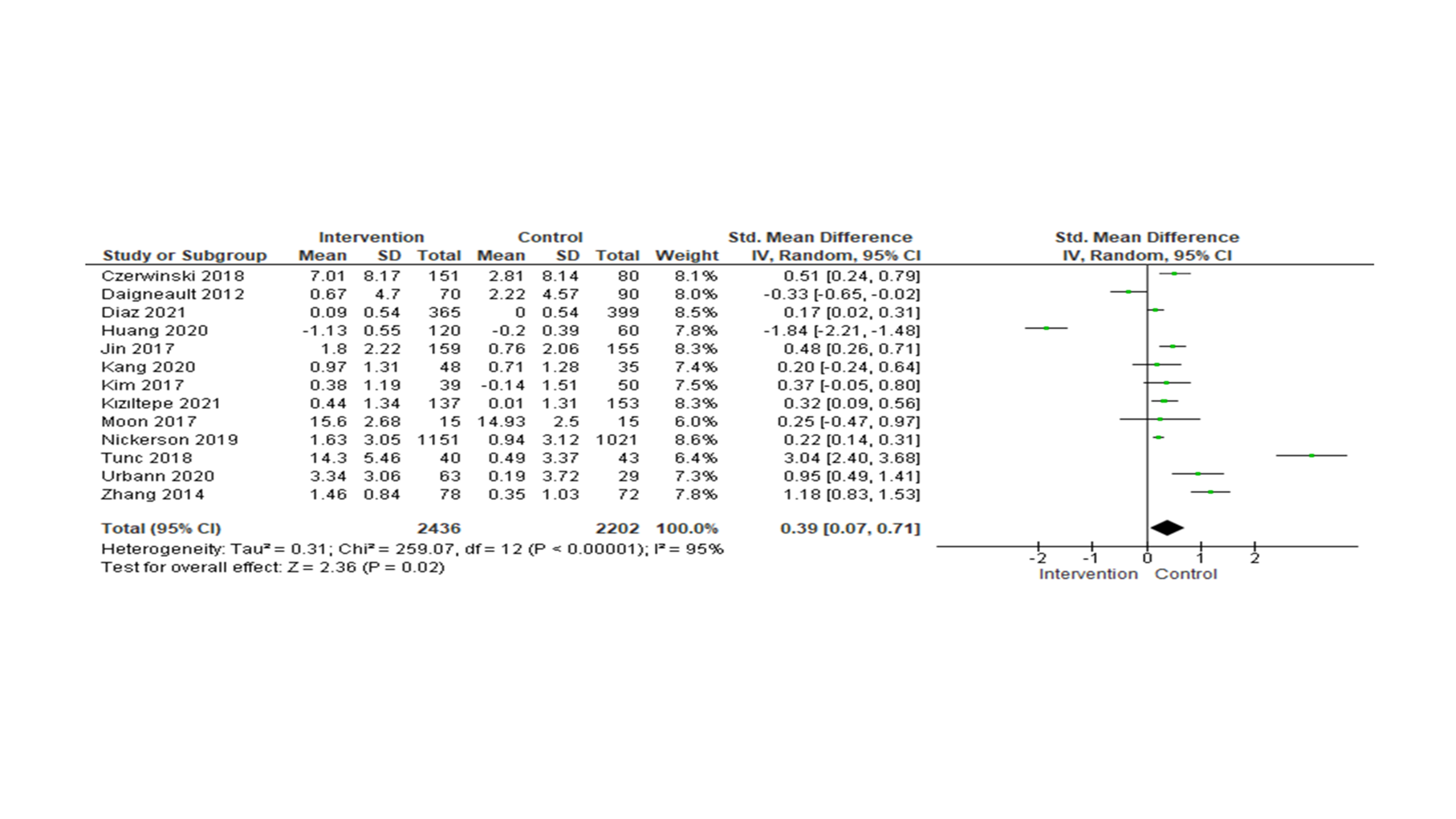

Supplement: Supplementary Figure 4 — Standardized mean difference of between-group intervention by skills. [file Image_4.TIF]

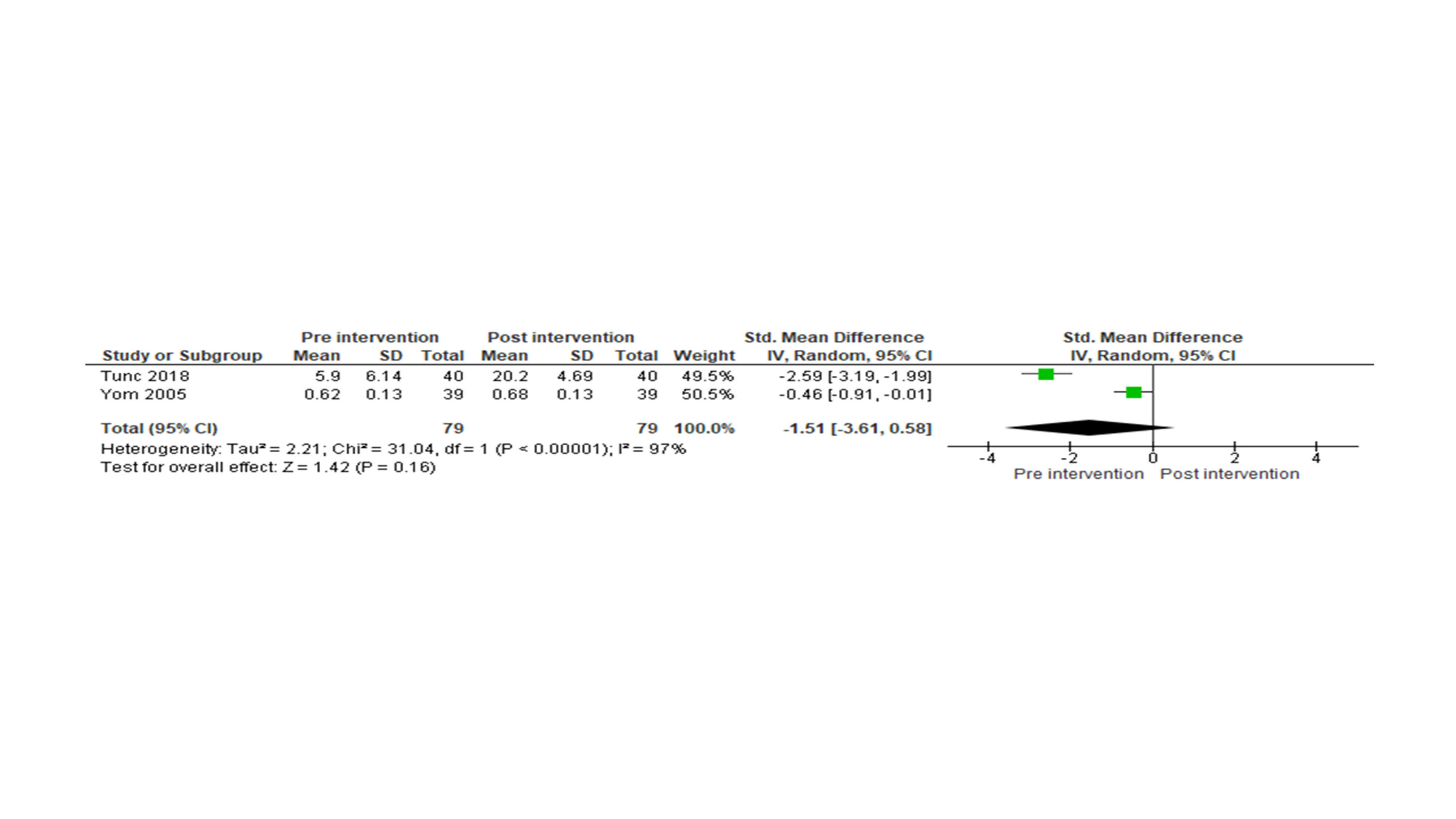

Supplement: Supplementary Figure 5 — Standardized mean difference of within-group intervention by attitude. [file Image_5.TIF]

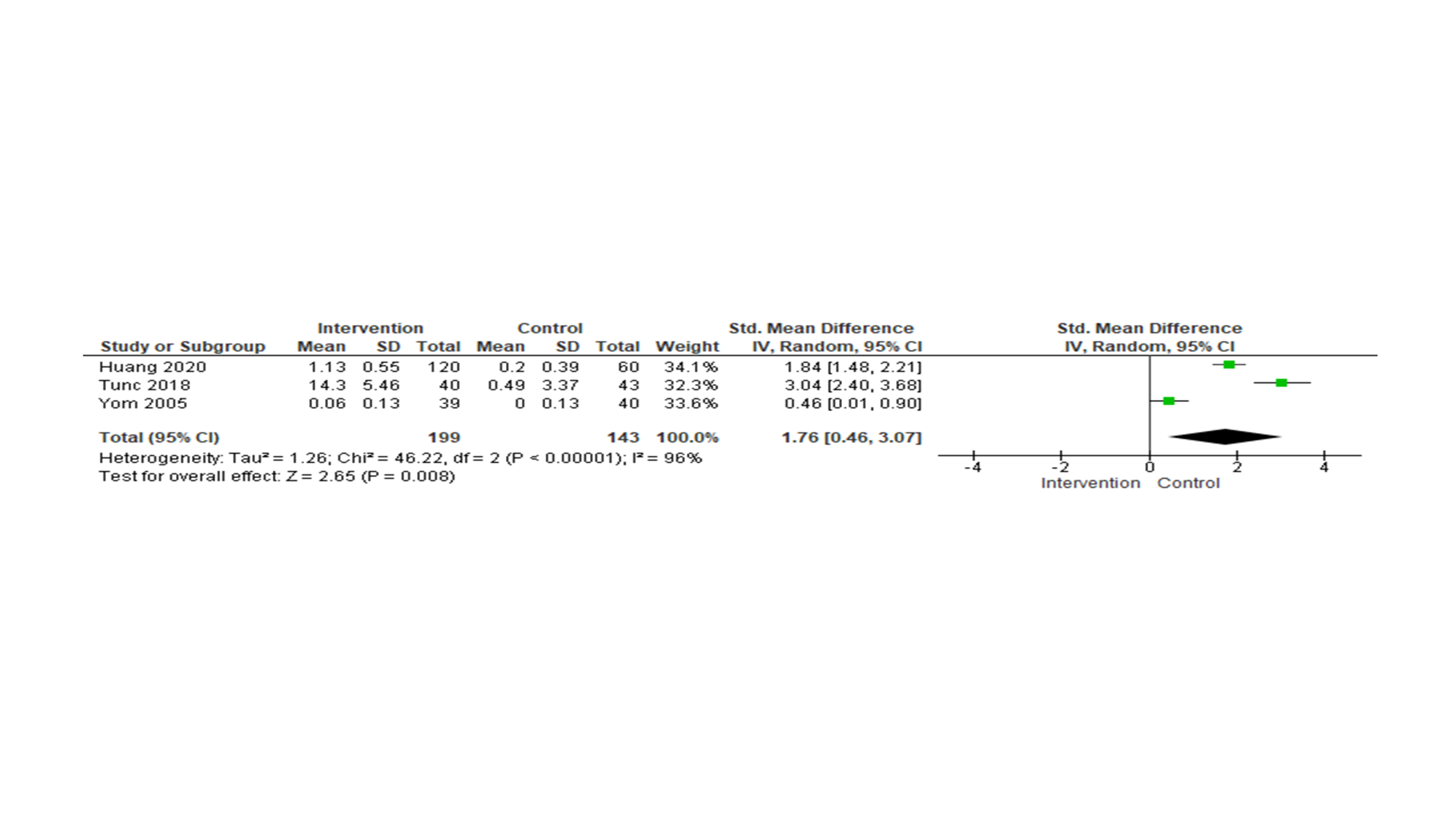

Supplement: Supplementary Figure 6 — Standardized mean difference of between-group intervention by attitude. [file Image_6.TIF]

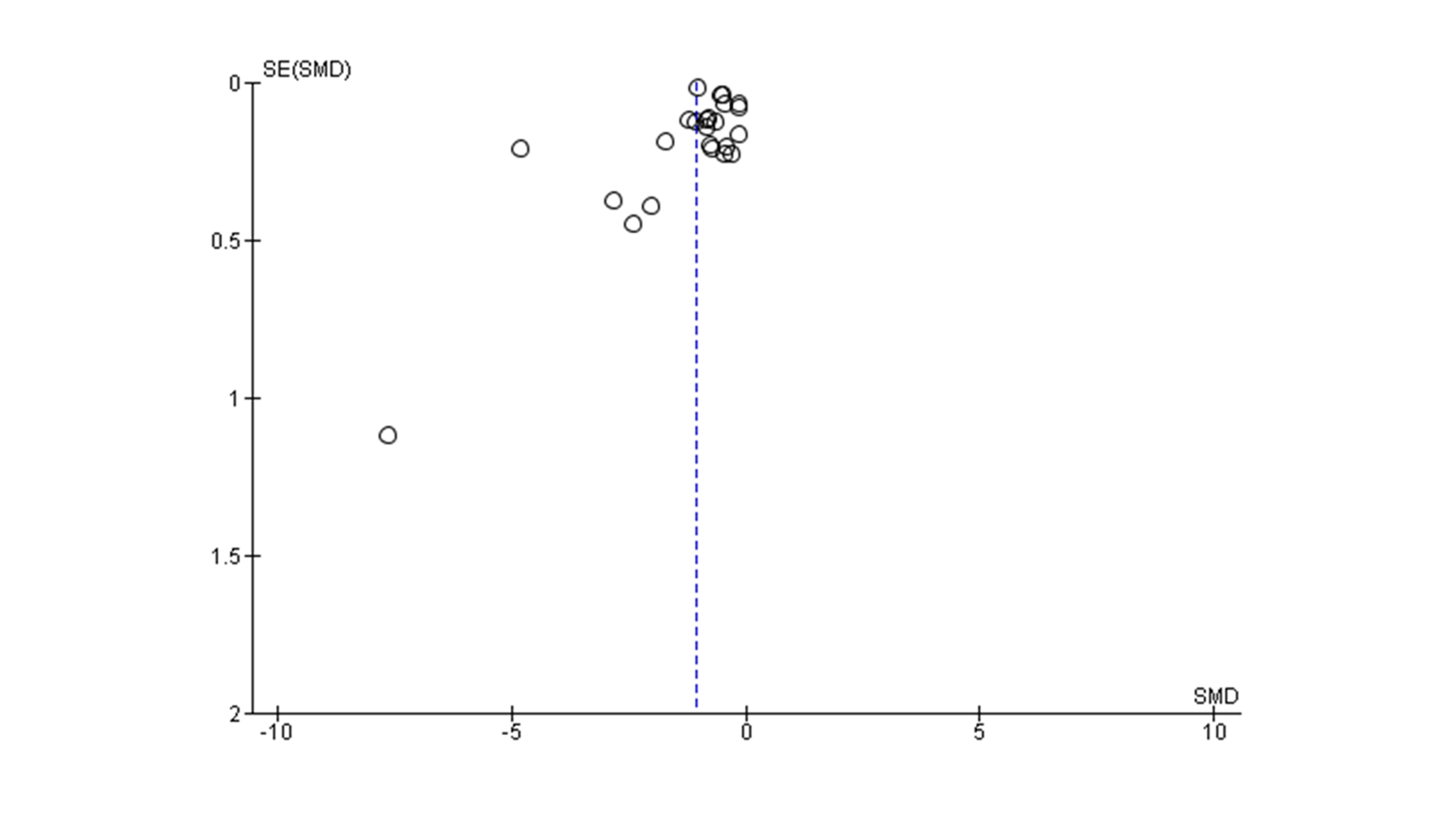

Supplement: Supplementary Figure 7 — Funnel plot of within-group comparison by knowledge. [file Image_7.TIF]

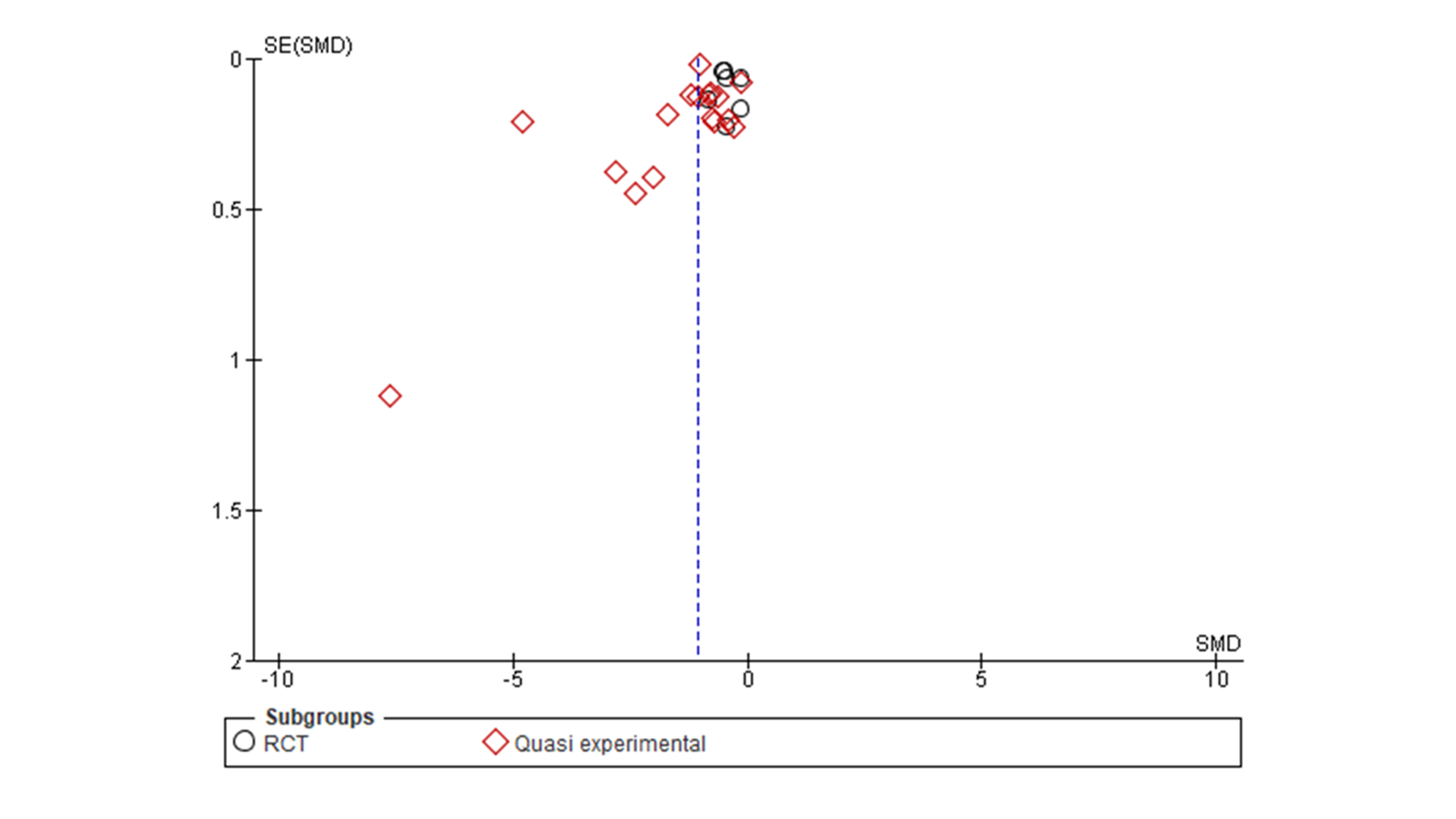

Supplement: Supplementary Figure 8 — Funnel plot of study design by knowledge. [file Image_8.TIF]

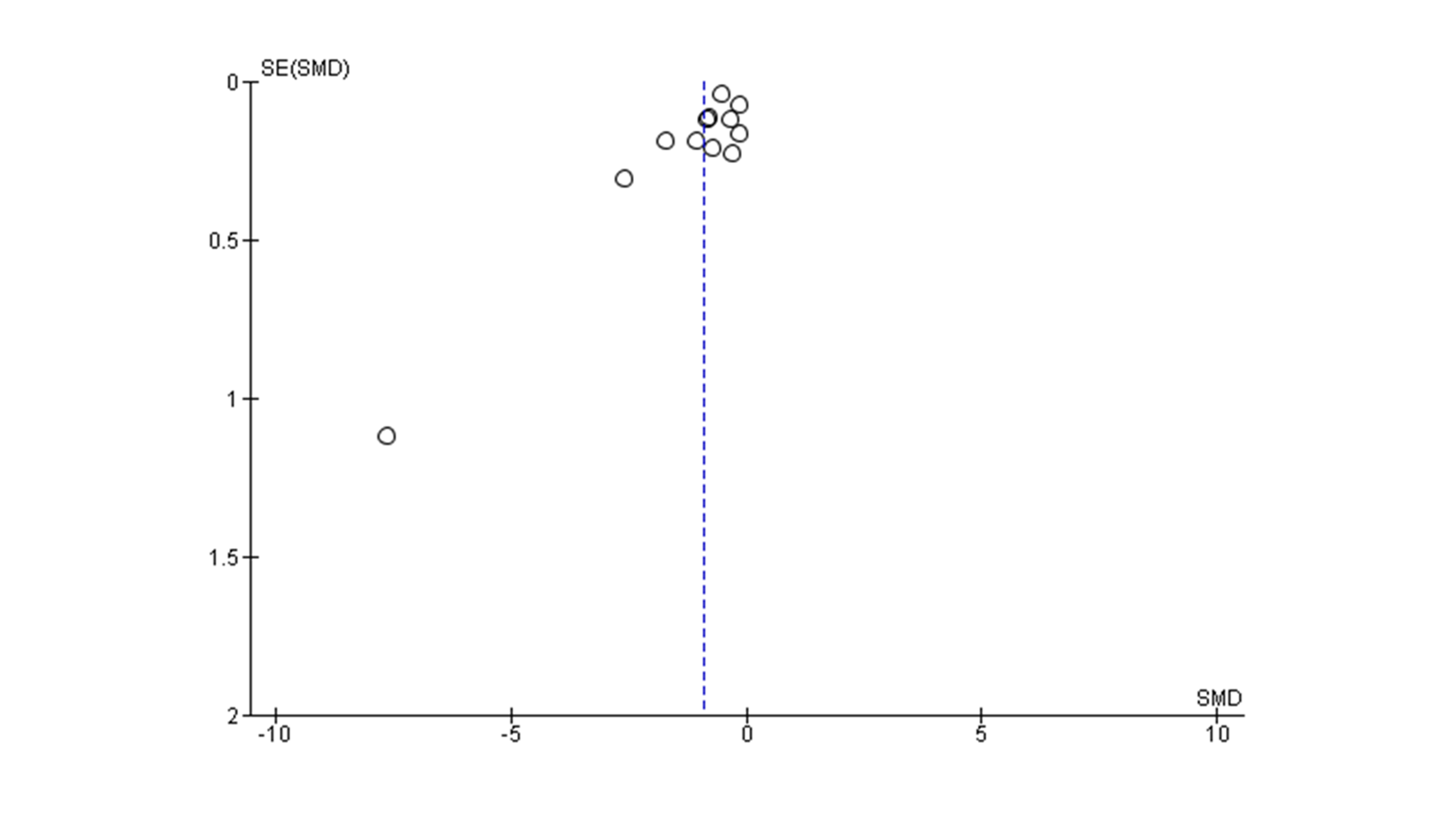

Supplement: Supplementary Figure 9 — Funnel plot of within-group comparison by skills. [file Image_9.TIF]

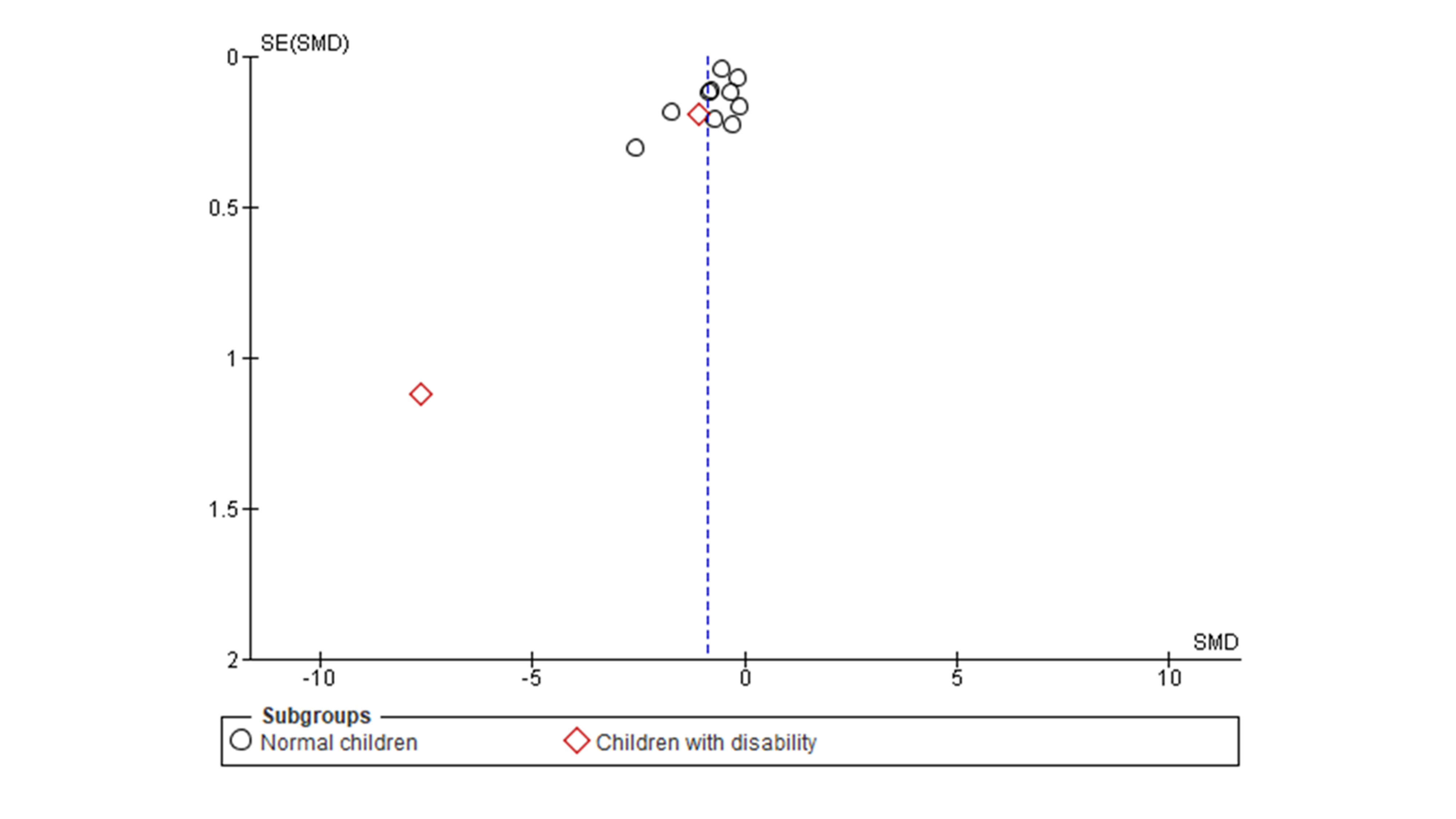

Supplement: Supplementary Figure 10 — Funnel plot of type of children by skills. [file Image_10.TIF]

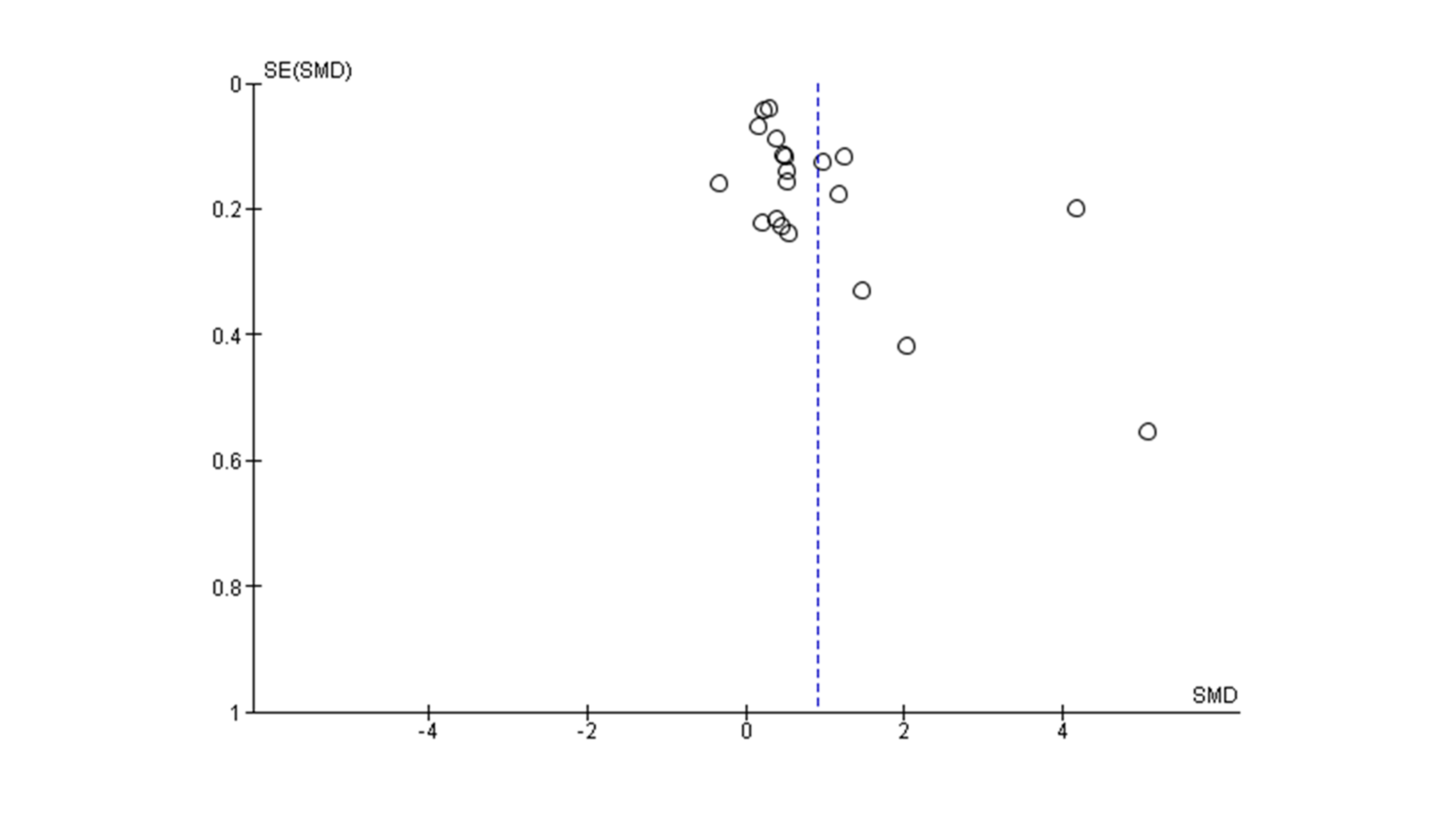

Supplement: Supplementary Figure 11 — Funnel plot of between-group comparison by knowledge. [file Image_11.TIF]

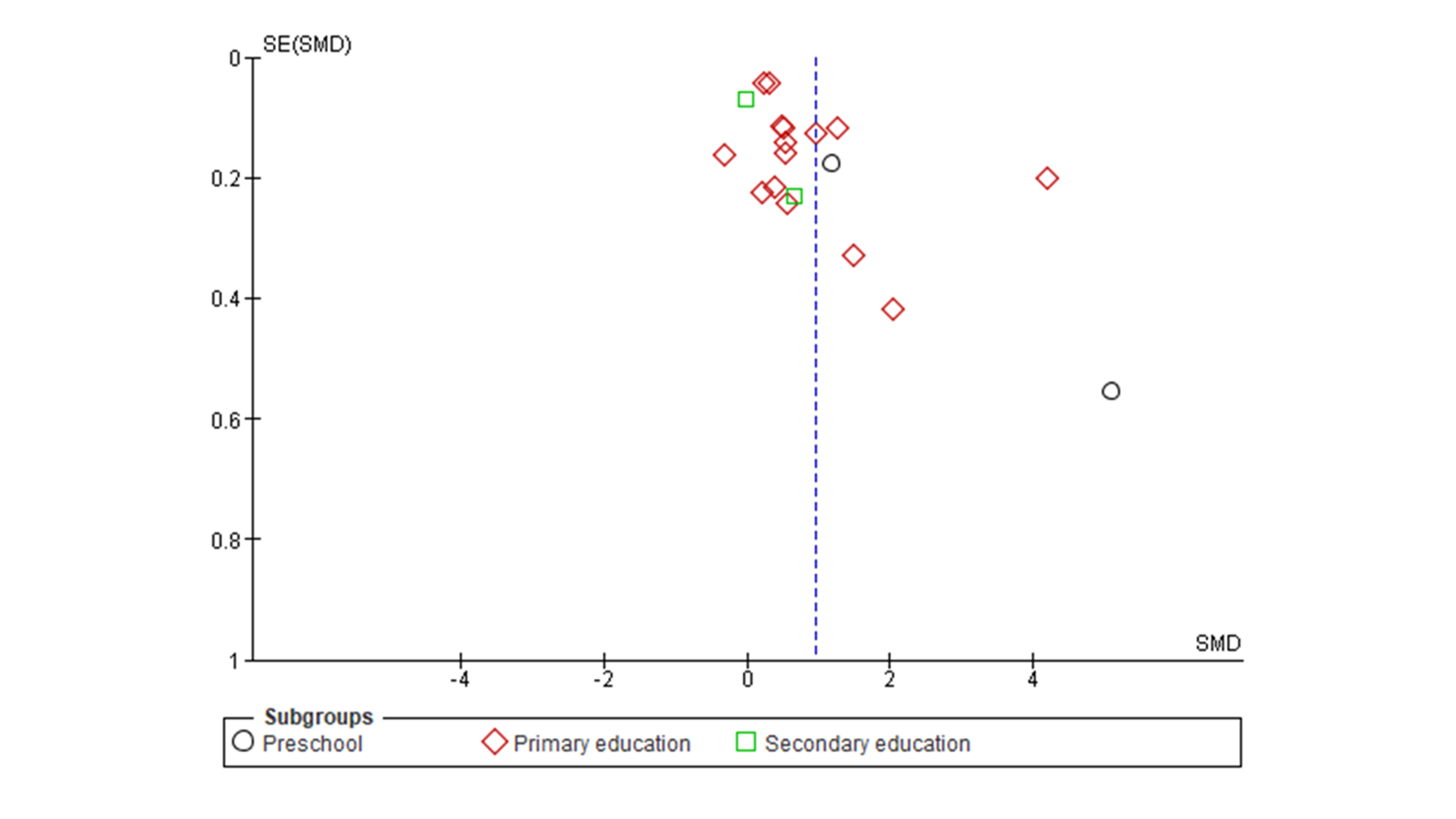

Supplement: Supplementary Figure 12 — Funnel plot of school level by knowledge. [file Image_12.TIF]

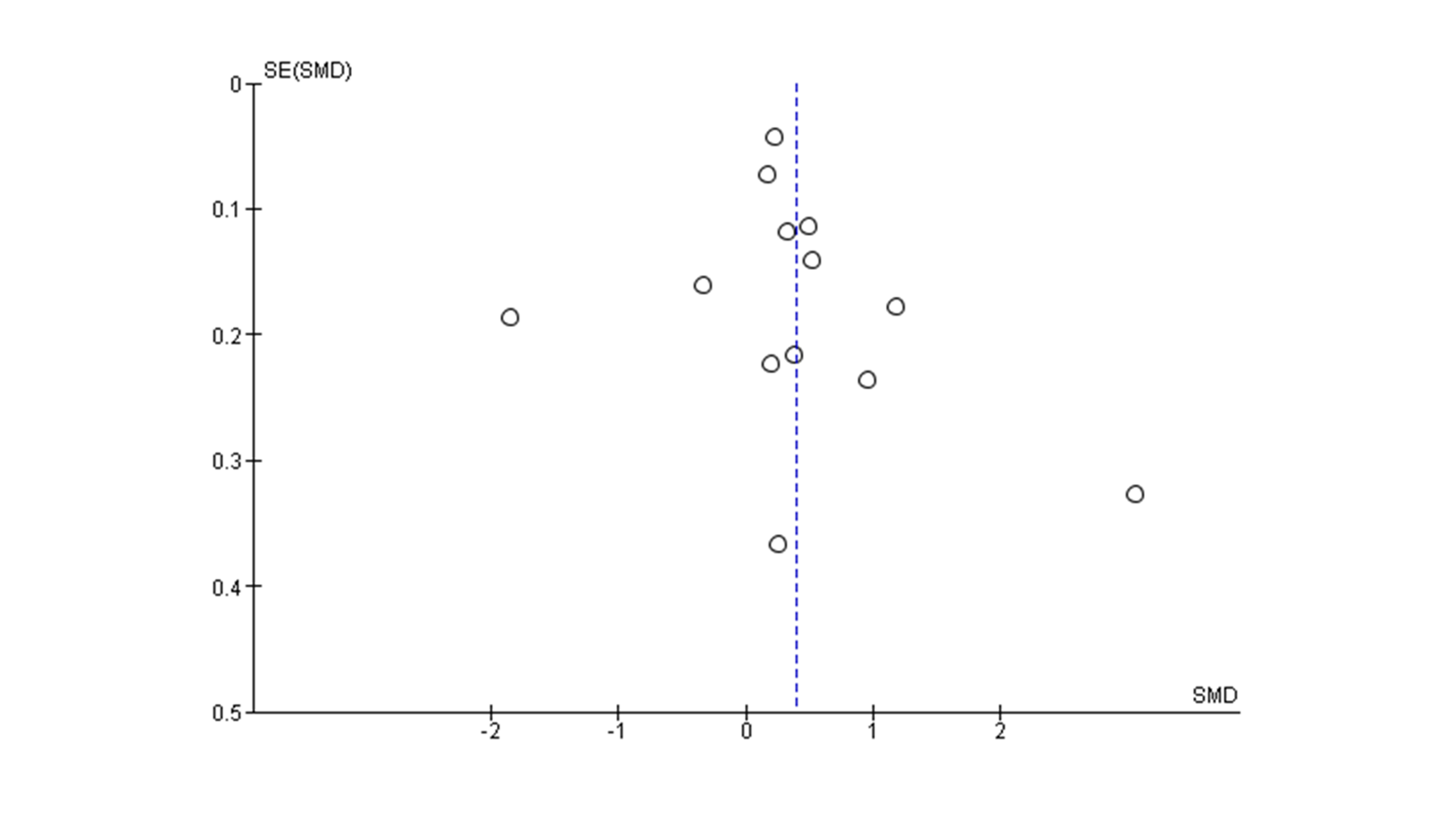

Supplement: Supplementary Figure 13 — Funnel plot of between-group comparison by skills. [file Image_13.TIF]
