# Supplementary material for: Effectiveness of school-based child sexual abuse intervention among school children in the new millennium era: Systematic review and meta-analyses
Source: Front Public Health. 2022 Jul 22;10:909254. doi: 10.3389/fpubh.2022.909254 (PMC9355675; doi:10.3389/fpubh.2022.909254)
Supplement: Supplementary Table 1 — Characteristics of the included study. [file Table_1.DOCX]

**Supplementary Table 1**: Characteristics of the included study

|  | **Author** | **Year** | **Study design** | **N** | **Program** | **Tools** | **Outcome measures** |
| --- | --- | --- | --- | --- | --- | --- | --- |
| 1 | Baker | 2013 | Quasi | 80 third grade students | My Body, My Boundaries curriculum | The standardized Children’s Knowledge of Abuse Questionnaire (CKAQ-R III; this was a shortened version of the original CKAQ) | Knowledge |
| 2 | Bustamante | 2019 | RCT | 4932 children aged 7–12 years | “I have the right to feel safe at all times” adapted | Del Campo Sanchez; "Children's Knowledge of Abuse Questionnaire-Revised" | Knowledge |
| 3 | Çeçen-eroǧul | 2013 | Quasi | 36 fourth grade students | A ‘preventing child sexual abuse psycho-educational training program’ | Good Touch Bad Touch Curriculum Test | Knowledge |
| 4 | Chamroonsawasdi | 2010 | Quasi | 256 primary school and 274 secondary school students. | Adapted WHO life skills development concepts | WHO life skills development concepts questionnaires | Attitude |
| 5 | Czerwinski | 2018 | Quasi | 291 third grade students | IGEL program | CKAQ-R III, a case vignette describing a situation with a child, What-If Situations Test | Knowledge, skills |
| 6 | Daigneault | 2012 | RCT | 160 first and third grades | The ESPACE workshop | Children Knowledge of Abuse Questionnaire (CKAQ-R), What If Situation Test (WIST), the nine-item Vaux Social Support Record, the seven-item empathic concern subscale of the Interpersonal Reactivity Index, The five-item Self-Efficacy questionnaire from the Teen Conflict Survey | Knowledge, skills |
| 7 | Dake | 2003 | Quasi | 341 third- grade students | Modified The child abuse prevention curriculum | The 26-item instrument. | Knowledge |
| 8 | Diaz | 2021 | RCT | 887 students grade 9 through 12 | Childhelp Speak Up Be Safe (CHSUBS) | 16-child maltreatment knowledge items, six questions about the RESIST strategies: Run, Escape, Scream, Ignore, Stay Away, and Tell. | Knowledge and skills |
| 9 | Edwards | 2020 | Quasi | 48 grade 4/5 American Indian children | Impower a 12-hour curriculum | The Children’s Knowledge of Abuse Questionnaire (CKAQ) | Knowledge, skills |
| 10 | Huang | 2020 | RCT | 180 preschool children | CSA-prevention picture book | CSA-prevention picture book, Preschool Attitude Scale, “What if” Situations Test (WIST), | Attitude, skills, |
| 11 | Jin | 2017 | Quasi | 484 primary school children | The curriculum based on previous studies and the personal safety part of Body Safe Training (BST) | Adapted previous studies questionnaire and the personal safety part of Body Safe Training (BST) | Knowledge and skills |
| 12 | Kang | 2020 | Quasi | 83 students grade 5 | Hybrid Application (CSAPE-H) | CSAPE-H | Knowledge, skills |
| 13 | Kim | 2017 | Quasi | 89 fifth-grade elementary school students | C-SAPE program | This 10-item tool was designed based on a health textbook, six multiple-choice items that evaluated the behaviors to protect themselves and the coping skills in various situations | Knowledge and skills |
| 14 | Kiziltepe | 2021 | Quasi | 290 preschool-aged children | “Mika” CSA prevention program | Child Sexual Abuse Knowledge Form, Self-protection Skill Form, | Knowledge and skills |
| 15 | Moon | 2017 | Quasi | 45 Primary School Children | Sexual Abuse Prevention Mobile Application (SAP_MobAPP) | Mobile app-based SAP education, The textbook based education programs | Skills |
| 16 | Moreno-manso | 2014 | Quasi | 317 Primary School Children | The programme (Moreno, Sánchez & Alcántara, 2006) | “The house in the forest” story | Knowledge |
| 17 | Muller | 2014 | Quasi | 286 elementary school children | “Cool and Safe” program | 9 items of Children's Knowledge of Abuse Questionnaire, four secure behaviors, 5 items of the Domain Specific Anxiety Questionnaire for Children, the Emotion Awareness Questionnaire, | Knowledge |
| 18 | Nickerson | 2019 | RCT | 2172 elementary school children | The Second Step Child Protection Unit (CPU) | The Children’s Knowledge of Abuse Questionnaire - Revised (CKAQ) and the Personal Safety Questionnaire (PSQ), “What If” Situation Test-III-R (WIST-R) | Knowledge, safety, skills, |
| 19 | Orak | 2021 | Quasi | 72 grade 4 students | Psychoeducation about sexual abuse. | Good Touch Bad Touch Curriculum Test” | Knowledge |
| 20 | Ozgun | 2021 | Quasi | 58 preschool children | Sexuality education program | “Questionnaire of Sexuality Knowledge Level” and “Personal Information Form” | Knowledge |
| 21 | Smothers | 2011 | Quasi | 132 grade 5-12 students | A Sexual Assault Primary Prevention Model | Sexual assault and attitudes questionnaire | Knowledge |
| 22 | Thompson | 2021 | RCT | 539 elementary school children | Play it Safe! | Play it Safe! | Knowledge |
| 23 | Tunc | 2018 | Quasi | 83 preschool children | CSA prevention programs | “Demographic Data Collection Form”, “What If Situations Test (WIST),” and the “Body Safety Training Program (BST) | Knowledge, skills and attitude |
| 24 | Tutty | 2020 | Quasi | 6198 elementary school children | “Who Do You Tell?” | The Children's Knowledge of Abuse Questionnaire-Revised | Knowledge |
| 25 | Urbann | 2020 | Quasi | 92 deaf and hard of hearing (DHH) primary school children | “STARK mit SAM” (Strong with Sam, SmS) | The questions of topics body (22 items), feelings (11 items), touches (4 items), and secrets and getting help (4 items for both topics). Additionally, the What If Situation Test (WIST) | Knowledge |
| 26 | Warraitch | 2020 | Quasi | 15 children with intellectual disabilities | A Child Sexual Abuse Prevention Intervention (CSAPI) | A child sexual abuse prevention knowledge and skills questionnaire | Knowledge and skills |
| 27 | Weeks | 2021 | RCT | 2414 elementary students at pre-test and 2260 at post-test | The MBF Child Safety Matters® (CSM) curriculum | Florida CSM evaluation | Knowledge |
| 28 | Yom | 2005 | RCT | 79 middle school male students | A CD-ROM titled Educational Program for the Prevention of Sexual Violence | A CD-ROM titled Educational Program for the Prevention of Sexual Violence was used for the intervention. The instrument contains 32 true-false items that measure knowledge level and 20 items comprising a four-point Likert-type scale that measure the attitude to sexual violence. Knowledge and Attitude of Sexual Violence Questionnaire (KASVQ) | Knowledge and attitude |
| 29 | Zhang | 2014 | Quasi | 150 Chinese preschool children | The Body Safe Training (BST) program | What If’’ Situations Test (WIST), The Personal Safety Questionnaire (PSQ) | Skills, knowledge |
