# Supplementary material for: Effectiveness of school-based child sexual abuse intervention among school children in the new millennium era: Systematic review and meta-analyses
Source: Front Public Health. 2022 Jul 22;10:909254. doi: 10.3389/fpubh.2022.909254 (PMC9355675; doi:10.3389/fpubh.2022.909254)
Supplement: Supplementary Table 2 — Assessment risk of bias by ROB-2. [file Table_2.DOCX]

**Supplementary Table 2**: Assessment risk of bias by ROB-2

| Risk-of-bias judgement (ROB-2) | Randomization process | Effect of assignment to intervention | Effect of adhering to intervention | Missing outcome data | Measurement of the outcome | Selection of the reported result | Overall risk of bias |
| --- | --- | --- | --- | --- | --- | --- | --- |
| Bustamante 2019 | low | low | some concern | low | low | high | low |
| Daigneault 2012 | low | low | some concern | low | low | high | low |
| Diaz 2021 | low | low | some concern | low | low | high | low |
| Huang 2020 | low | low | some concern | low | low | high | low |
| Nickerson 2019 | low | low | some concern | low | low | high | low |
| Thompson 2021 | low | low | some concern | low | low | high | low |
| Weeks 2021 | low | low | some concern | low | low | high | low |
| Yom 2005 | low | low | some concern | low | low | high | low |
