# Supplementary material for: Effectiveness of school-based child sexual abuse intervention among school children in the new millennium era: Systematic review and meta-analyses
Source: Front Public Health. 2022 Jul 22;10:909254. doi: 10.3389/fpubh.2022.909254 (PMC9355675; doi:10.3389/fpubh.2022.909254)
Supplement: Supplementary Table 3 — Assessment risk of bias by ROBINS-I. [file Table_3.DOCX]

**Supplementary Table 3**: Assessment risk of bias by ROBINS-I

| Risk of bias judgement (ROBINS-I) | Bias due to confounding | Bias in selection of participants into the study | Bias in classification of interventions | | Bias due to deviations from intended interventions | Bias due to missing data | Bias in measurement of outcomes | Bias in selection of the reported result | | Overall bias |
| --- | --- | --- | --- | --- | --- | --- | --- | --- | --- | --- |
| Baker 2013 | low | low | | low | low | low | moderate | | low | low |
| Çeçen-Eroǧul 2013 | low | low | | low | low | low | moderate | | low | low |
| Chamroonsawasdi 2010 | low | low | | low | low | low | moderate | | low | low |
| Czerwinski 2018 | low | low | | low | low | low | moderate | | low | low |
| Dake 2003 | low | low | | low | low | low | moderate | | low | low |
| Edwards 2020 | low | low | | low | low | low | moderate | | low | low |
| Jin 2017 | low | low | | low | low | low | moderate | | low | low |
| Kang 2020 | low | low | | low | low | low | moderate | | low | low |
| Kim 2017 | low | low | | low | low | low | moderate | | low | low |
| Kızıltepe 2021 | low | low | | low | low | low | moderate | | low | low |
| Moon 2017 | low | low | | low | low | low | moderate | | low | low |
| Moreno-Manso 2014 | low | low | | low | low | low | moderate | | low | low |
| Müller 2014 | low | low | | low | low | low | moderate | | low | low |
| Orak 2021 | low | low | | low | low | low | moderate | | low | low |
| Ozgun 2021 | low | low | | low | low | low | moderate | | low | low |
| Smothers 2011 | low | low | | low | low | low | moderate | | low | low |
| Tunc 2018 | low | low | | low | low | low | moderate | | low | low |
| Tutty 2020 | low | low | | low | low | low | moderate | | low | low |
| Urbann 2020 | low | low | | low | low | low | moderate | | low | low |
| Warraitch 2021 | low | low | | low | low | low | moderate | | low | low |
| Zhang 2014 | low | low | | low | low | low | moderate | | low | low |
