# Supplementary material for: Effectiveness of school-based child sexual abuse intervention among school children in the new millennium era: Systematic review and meta-analyses
Source: Front Public Health. 2022 Jul 22;10:909254. doi: 10.3389/fpubh.2022.909254 (PMC9355675; doi:10.3389/fpubh.2022.909254)
Supplement: Supplementary Table 4 — The quality of evidence for the within-group comparison according to GRADEpro methodology. [file Table_4.DOCX]

**Supplementary Table 4**: The quality of evidence for the within group comparison according to the GRADEpro methodology

| **Outcomes** | **№ of participants (studies) Follow-up** | **Certainty of the evidence (GRADE)** | **Relative effect (95% CI)** | **Anticipated absolute effects** | |
| --- | --- | --- | --- | --- | --- |
|  |  |  |  | **Risk with** | **Risk difference with within group** |
| Knowledge | 20022 (24 RCTs) | ⨁⨁⨁⨁ High | - | - | SMD **1.06 lower** (1.29 lower to 0.84 lower) |
| Skills | 4632 (12 RCTs) | ⨁⨁⨁⨁ High^a^ | - | - | SMD **0.91 lower** (1.2 lower to 0.61 lower) |
| Attitude | 158 (2 RCTs) | ⨁⨁⨁⨁ High | - | - | SMD **1.51 lower** (3.61 lower to 0.58 higher) |
| Study design | 20069 (24 RCTs) | ⨁⨁⨁⨁ High | - | - | SMD **1.06 lower** (1.29 lower to 0.84 lower) |
| Study design - RCT | 6737 (7 RCTs) | ⨁⨁⨁⨁ High | - | - | SMD **0.44 lower** (0.58 lower to 0.31 lower) |
| Study design - Quasi experimental | 13332 (17 RCTs) | ⨁⨁⨁⨁ High | - | - | SMD **1.43 lower** (1.78 lower to 1.07 lower) |
| Type of children | 4666 (12 RCTs) | ⨁⨁⨁⨁ High^a^ | - | - | SMD **0.9 lower** (1.2 lower to 0.61 lower) |
| Type of children - Normal children | 4510 (10 RCTs) | ⨁⨁⨁⨁ High | - | - | SMD **0.76 lower** (1.04 lower to 0.49 lower) |
| Type of children - Children with disability | 156 (2 RCTs) | ⨁⨁⨁⨁ High | - | - | SMD **4.27 lower** (10.69 lower to 2.15 higher) |

^a^ The funnel plot was asymmetry and the Egger's test was significant
