# Supplementary material for: Effectiveness of school-based child sexual abuse intervention among school children in the new millennium era: Systematic review and meta-analyses
Source: Front Public Health. 2022 Jul 22;10:909254. doi: 10.3389/fpubh.2022.909254 (PMC9355675; doi:10.3389/fpubh.2022.909254)
Supplement: Supplementary Table 5 — The quality of evidence for the between-group comparison according to GRADEpro methodology. [file Table_5.DOCX]

**Supplementary Table 5**: The quality of evidence for the between group comparison according to the GRADEpro methodology

| **Outcomes** | **№ of participants (studies) Follow-up** | **Certainty of the evidence (GRADE)** | **Relative effect (95% CI)** | **Anticipated absolute effects** | |
| --- | --- | --- | --- | --- | --- |
|  |  |  |  | **Risk with** | **Risk difference with between groups** |
| Knowledge | 8740 (20 RCTs) | ⨁⨁⨁⨁ High^a^ | - | - | SMD **0.9 higher** (0.63 higher to 1.18 higher) |
| Skills | 4638 (13 RCTs) | ⨁⨁⨁⨁ High | - | - | SMD **0.39 higher** (0.07 higher to 0.71 higher) |
| Attitude | 342 (3 RCTs) | ⨁⨁⨁⨁ High | - | - | SMD **1.76 higher** (0.46 higher to 3.07 higher) |
| School level | 8210 (19 RCTs) | ⨁⨁⨁⨁ High^a^ | - | - | SMD **0.94 higher** (0.64 higher to 1.24 higher) |
| School level - Preschool | 208 (2 RCTs) | ⨁⨁⨁⨁ High | - | - | SMD **3.08 higher** (0.72 lower to 6.89 higher) |
|  |  |  |  |  |  |
| School level - Primary school | 7090 (15 RCTs) | ⨁⨁⨁⨁ High | - | - | SMD **0.84 higher** (0.51 higher to 1.18 higher) |
| School level - Secondary school | 912 (2 RCTs) | ⨁⨁⨁⨁ High | - | - | SMD **0.28 higher** (0.4 lower to 0.95 higher) |

^a^ The funnel plot was asymmetry and the Egger's test was significant
